# Human Mitochondrial Control Region and mtGenome: Design and Forensic Validation of NGS Multiplexes, Sequencing and Analytical Software

**DOI:** 10.3390/genes12040599

**Published:** 2021-04-19

**Authors:** Cydne L. Holt, Kathryn M. Stephens, Paulina Walichiewicz, Keenan D. Fleming, Elmira Forouzmand, Shan-Fu Wu

**Affiliations:** Verogen Inc., San Diego, CA 92121, USA; kstephens@verogen.com (K.M.S.); paulinka@gmail.com (P.W.); kfleming@verogen.com (K.D.F.); eforouzmand@verogen.com (E.F.); swu@verogen.com (S.-F.W.)

**Keywords:** mtDNA, mitochondria, forensic, validation, Scientific Working Group on DNA Analysis Methods (SWGDAM), massively parallel sequencing, next generation sequencing, ForenSeq, MiSeq, sequencing by synthesis

## Abstract

Forensic mitochondrial DNA (mtDNA) analysis conducted using next-generation sequencing (NGS), also known as massively parallel sequencing (MPS), as compared to Sanger-type sequencing brings modern advantages, such as deep coverage per base (herein referred to as read depth per base pair (bp)), simultaneous sequencing of multiple samples (libraries) and increased operational efficiencies. This report describes the design and developmental validation, according to forensic quality assurance standards, of end-to-end workflows for two multiplexes, comprised of ForenSeq mtDNA control region and mtDNA whole-genome kits the MiSeq FGx^TM^ instrument and ForenSeq universal analysis software (UAS) 2.0/2.1. Polymerase chain reaction (PCR) enrichment and a tiled amplicon approach target small, overlapping amplicons (60–150 bp and 60–209 bp for the control region and mtGenome, respectively). The system provides convenient access to data files that can be used outside of the UAS if desired. Studies assessed a range of environmental and situational variables, including but not limited to buccal samples, rootless hairs, dental and skeletal remains, concordance of control region typing between the two multiplexes and as compared to orthogonal data, assorted sensitivity studies, two-person DNA mixtures and PCR-based performance testing. Limitations of the system and implementation considerations are discussed. Data indicated that the two mtDNA multiplexes, MiSeq FGx and ForenSeq software, meet or exceed forensic DNA quality assurance (QA) guidelines with robust, reproducible performance on samples of various quantities and qualities.

## 1. Introduction

Human mitochondrial haplotype testing (mitotyping) is valuable in forensically relevant scenarios [1,2,3,4,5,6,7,8,9]. While next-generation sequencing (NGS) has been quite commonly used in recent years in disciplines outside of the forensic sciences, Sanger-based sequencing on capillary electrophoresis instruments has been the most employed method for analyses of samples of forensic significance, such as historical or ancient human skeletal and dental remains, rootless hair shafts and biological materials that have undergone extreme environmental insults as well as for investigative matters of maternal lineage or genetic insights gleaned from haplogroup assignment [10,11,12,13,14]. The resilience of mtDNA in forensic samples is attributed to the high copy number per cell relative to the nuclear genome and physical properties, including its circular organization. Microscopic hair examinations in forensic settings can sometimes be strengthened by pairing with mtDNA sequencing when attempting to exclude potential sources [15].

Forensic mtDNA analyses by Sanger sequencing largely focused on two hypervariable regions (HVI, HVII) within the control region (CR, approximately 1122 bp), so-called as it contains the mitochondrial origin of replication and transcription. This small portion of the mitochondrial genome (mtGenome, approximately 16,569 bp) has been most explored due to its noncoding nature and the level of effort required to generate 1–2x sequencing coverage with chain termination chemistry. As mtDNA analysis continues the transition to the less labor-intensive and deeper read depth produced by next-generation sequencing (NGS), also known as massively parallel sequencing (MPS), not only is CR analysis more approachable, but analysis of complete mtGenomes is now routinely achieved [16,17,18,19,20,21,22,23,24,25,26,27,28,29,30,31]. Research efforts can now be propelled further by these complete data sets, and meaningfully refined haplogroups assignments can be generated relative to data from the control region alone [19,32,33,34].

**Figure 1:**
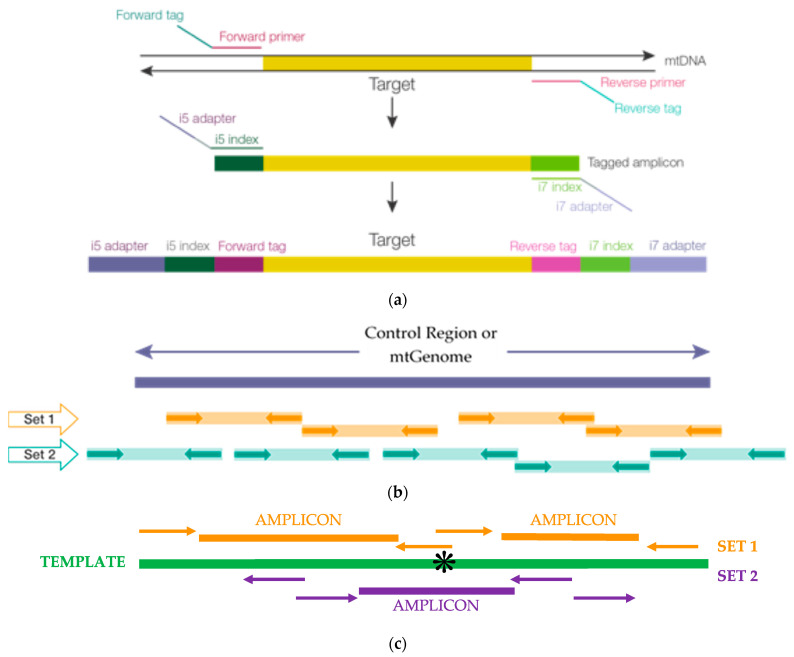
Schematic of ForenSeq mtDNA control region and mtDNA whole-genome library preparation design and workflow. (**a**) PCR-based target enrichment and tagging create DNA templates consisting of regions of interest flanked by universal primer sequences. Index adapters then attach to the tags. Resultant paired-end, dual-indexed, tiled libraries are amplified, purified, and pooled into one tube for MiSeq FGx sequencing and analysis with ForenSeq UAS or other software, (**b**) ForenSeq protocol divides each DNA sample into two PCRs with separate primer sets (set 1, set 2) in a tiled strategy that promotes efficient amplification of overlapping amplicons to allow complete coverage and reduces unintended byproducts, (**c**) the two-PCR approach can facilitate confirmation of variant(s) that reside under a primer: when a primer-binding site mutation exists under a primer in one primer set (asterisk under a set 1 primer in this diagram), then that variant can be reliably detected in amplicons extended from the companion primer set (set 2). Alternative approaches can more frequently produce ambiguity regarding primer sequences versus variants.

This report describes the design and developmental validation according to forensic quality assurance standards [1,2] of two multiplexes, provided as ForenSeq™ mtDNA control region and mtDNA whole-genome kits, the MiSeq Forensic Genomics (FGx^TM^) instrument and ForenSeq universal analysis software (UAS) 2.0/2.1. ForenSeq chemistry in these mtDNA kits is based on a previously validated system that targets nuclear loci, the ForenSeq DNA signature prep kit [35] and uses PCR enrichment (no ligation) with a tiled amplicon approach that generates small overlapping amplicons (60–150 bp for control region (avg 118), 60–209 bp (avg 131) for mtGenome) to achieve complete coverage of the targets, including under primer-binding sites as often as possible, and reliable performance with highly degraded samples. An average of 17 bp overlap (3 bp overlap minimum) between adjacent amplicons mitigates data loss in end-of-read trimming during data analyses. These validated assays target 18 amplicons from 122 primers (control region) and 245 amplicons from 663 primers (mtGenome) to effectively anneal and extend across human sequence variations (Figure 1) then sequenced on the MiSeq FGx^®^ instrument. The MiSeq FGx employs sequencing-by-synthesis (SBS), the most accurate and widely used NGS available [36,37,38,39,40,41]. ForenSeq UAS 2.0/2.1 automatically generates mtDNA variant calls as compared to the revised Cambridge reference sequence (rCRS) and using standardized nomenclature accepted by the global forensic science community [42]. This software was designed for forensic use and provides a relatively flat workflow as compared to research tools: it does not require multiple programs to deliver results and includes tools and reports intended to address casework and databasing needs. The system provides convenient access to files that can be used outside of the UAS if desired, especially in research or custom workflows. Performance and limitations of the end-to-end workflow in generating control region and mtGenome data were investigated in studies, including, but not limited to mock casework samples (buccal, rootless hairs, dental, skeletal remains), concordance of control region typing between the two kits and as compared to orthogonal data, assorted sensitivity studies, repeatability, reproducibility, two-person DNA mixtures, and performance windows for thermal cycling parameters and critical PCR reagents as well as NGS-specific forensic studies. Results described herein demonstrate that the two mtDNA multiplexes, MiSeq FGx and ForenSeq UAS, meet or exceed forensic DNA quality assurance guidelines with robust, reliable, and reproducible performance on samples of various quantities and qualities.

## 2. Materials and Methods

### 2.1. Primer Design and Placement, Population Studies: mtDNA Control Region and Mitochondrial Genome (mtGenome) Multiplexes

PCR primers were designed using internal expertise, Primer3 and iterative testing [43,44,45]. Degenerate bases designed into the primers were based on variants with frequencies >1%, as reported in MitoMap [46] among 48,882 full-length mtGenome sequences that represent 33 macro-haplogroups known to date (see Section 3.6). Target enrichment utilized a pool of 122 primers targeting 18 amplicons for the control region multiplex and 663 primers targeting 245 amplicons for the whole mtGenome multiplex, including 116 and 237 oligonucleotides with one or more degenerate (wobbles) base(s) for CR and mtG multiplexes, respectively. Primer placement was tiled using an overlapping strategy that promotes complete coverage (Figure 1). Adjacent amplicons overlap on average by 17 bp (3 bp overlap minimum) to prevent data loss when primers are bioinformatically trimmed. Resultant amplicon length ranges (for HL60 ForenSeq^TM^ positive control DNA) were 60 to 150 bp (mean length 118 bp) for control region multiplex and 60 to 209 bp (mean length 131 bp) for the mtGenome multiplex. Seventy-one DNA samples from four major population groups and 15 subtrees (B, C, D, F, H, J, K, L, M, N, R, T, U, W, X) were sequenced from dental and skeletal remains (Table 1, *n* = 16), buccal and hair samples (*n* = 6, see Section 3.1.2) and 1000 Genomes Project DNA samples (Coriell Institute for Medical Research, Camden, NJ, USA; *n* = 49, see Section 3.5.2) in a small, diverse population study resulting in 63 unique haplotypes. Expanded ancestries for Coriell samples are shown in Supplementary Table S1.

### 2.2. ForenSeq Positive Control, Human DNA Samples, Mock Casework Samples

HL60 (Millipore-Sigma, St. Louis, MO, USA), the positive gDNA control sample supplied in each ForenSeq mtDNA kit and supported by the ForenSeq universal analysis software (UAS) with positive control metrics, served as the positive amplification and library preparation control throughout these studies (please see “MiSeq FGx Sequencing” for human sequencing control). Additional standards and controls included the standard reference material (SRM) samples NIST SRM2392 (contains 9947A, CHR) and SRM2392-I (contains HL60—this is a different source than ForenSeq positive control HL60), from the National Institute of Standards and Technology (NIST, Gaithersburg, MD, USA) and 2800M gDNA (Promega Corporation, Madison, WI, USA), gDNA samples NA03798, NA10472, HG01204, NA18524 and 47 additional 1000 Genomes Project DNA samples (Coriell Institute for Medical Research, Camden, NJ, USA) (Supplemental Table S1). DNA extracts of mock casework samples (Table 1) consisted of five contemporary tooth samples (InnoGenomics Inc., New Orleans, LA, USA) extracted with the dental forensic kit (DFK^®^) [47], seven contemporary bone extracts provided by Dr. Rachel Houston (Department of Forensic Science, Sam Houston State University (SHSU), Huntsville, TX, USA) in conjunction with the willed body program at the Southeast Texas Applied Forensic Science Facility, and four DNA extracts from ancient bones of Eastern European origin provided by the laboratory of Dr. Mitchell Holland (Department of Biochemistry & Molecular Biology, Pennsylvania State University (PSU), State College, PA, USA). SHSU bone samples were recovered from cadavers (postmortem intervals range from approximately one to eight years (based on case numbers), stored frozen, thawed placed at SHSU’s Applied Anatomical Research Center (AARC) Outdoor Research Facility, subjected to various treatments (Table 1) and extracted for DNA using PrepFiler™ forensic DNA extraction kit (Thermo Fisher, Waltham, MA, USA) [48] for samples S1, S3, S4, S6 and S7 or demineralization protocol for bone samples S2 and S5 [49]. Dental samples and SHSU bone samples were amplified at 100 pg inputs; PSU bone samples were amplified at ~8000 mtDNA copies. Hair and buccal swabs were extracted with PrepFiler forensic DNA extraction kit (Thermo Fisher, Waltham, MA, USA), and the protocol described by Gallimore et al. was followed for hair samples [33].

### 2.3. Sensitivity and Mixture DNA Studies

Supplementary Table S2A summarizes the samples, gDNA inputs, replicate numbers, purification rounds (one or two), normalization methods (bead-based normalization (BBN) or manual quantification (QN)) and average reads per sample (see Section 3.2) for sensitivity studies. The library preparation protocols for ForenSeq mtDNA kits include two methods for library normalization with BBN intended for processing in a high-throughput environment (and may be automated) and QN intended for low-input samples (≤20 pg total input) or when handling samples of varying inputs (i.e., low and high input samples in the same batch). While QN may be used for high-throughput processing, it may be considered less efficient due to the hands-on time required.

Sensitivity was assessed using HG01204 and NA18524 Coriell DNAs serially diluted into molecular grade water for inputs of 50, 10, 5, 2.5, 1 or 0.5 pg of gDNA each into the two ForenSeq mtDNA control region amplifications, for a total of 100, 20, 10, 5, 2 or 1 pg input into the final libraries. HG01204 and NA18524 DNA dilution samples were amplified in duplicate along with four replicates each of 100 pg HL60 positive control gDNA and four negative amplification controls (purified water) for 32 total libraries. Libraries were normalized, using both bead-based (BBN) and manual quantification (QN) methods, and sequenced on two MiSeq FGx sequencing runs. A second sensitivity study was performed with HL60 serially diluted into molecular grade water for inputs of 5000, 500, 250, 100, 50, 25, 10, 5, 2.5, 1 or 0.5 pg of gDNA each into the two amplifications, for a total of 10,000, 1000, 500, 200, 100, 50, 20, 10, 5, 2 or 1 pg input into the final library. The HL60 DNA dilution samples were amplified using the ForenSeq mtDNA control region kit in quadruplicate with three negative amplification controls, normalized using QN, and sequenced on the MiSeq FGx with 12 and with 47 libraries (sample plexity). HL60 DNA was amplified in duplicate using the ForenSeq mtDNA whole-genome kit in each of two studies with inputs of 100, 50, 30, 20, 10, 5, 2 as follows: one set of libraries was purified using the one round of purification and normalized with BBN; a second set was purified with a second optional round of purification (using sample Purification Beads supplied in ForenSeq kits) and normalized with QN. The two sets of libraries were sequenced on separate sequencing runs with libraries 16. Variant calls, as analyzed relative to the revised Cambridge Reference sequence (rCRS) with ForenSeq UAS, were compared to variant calls from whole-genome sequencing (WGS) data [50,51] or results obtained using PowerSeq^®^ CRM nested system, Custom (Promega Corporation, Madison, WI, USA).

Supplementary Table S2B summarizes the samples, mixture ratios, gDNA inputs, replicate numbers, purification rounds (one or two) and normalization methods (BBN or QN) for mixture studies. Human genomic DNA mixtures were prepared by combining two gDNA samples (HL60 as a major donor, 2800M as a minor donor) amplifying with each ForenSeq mtDNA multiplex, at 100 pg and 5 pg total input gDNA, as follows: control region multiplex at ratios 1:3, 1:5 and 1:15 in duplicate; mtGenome multiplex at ratios 1:1, 1:3 and 1:9 in duplicate. Control region or mtGenome libraries were sequenced at 47 and 12 sample plexity, respectively. Expected minor and major expected ratios were calculated by determining the expected (published) HL60 variants and the read counts for the single-source samples that were used to create the DNA mixtures, in the fashion of Brandhagen et al. [26].

### 2.4. Repeatability and Reproducibility Studies

Supplementary Table S2C,D summarize the samples, gDNA inputs, replicate numbers, purification rounds (one or two) and normalization methods (BBN or QN) for repeatability studies and reproducibility studies, respectively. Repeatability and reproducibility studies were performed for the ForenSeq mtDNA control region and mtDNA whole-genome kits and software. Repeatability studies for the control region multiplex were performed by sequencing the same pool of 16 libraries on three MiSeq FGx instruments. Control region libraries were prepared in triplicate from 100 pg and 2 pg HL60 positive control gDNA and 2 negative amplification controls (purified water), using BBN and two many manufactured kits. Repeatability studies for the mtGenome multiplex were performed by sequencing the same pool of 16 libraries on three MiSeq FGx instruments. mtGenome libraries were prepared from one to replicate each of 20 pg and 2 pg HL60 positive control gDNA (following two rounds of purification and QN), from 100 pg and 2 pg HL60 positive control gDNA in duplicate, one replicates each prepared from 20 pg HL60 and from 100 pg each of samples HG02190, HG02215, HG02449, HG01497, NA12870, SRM 2392-I HL60, 9947A, CHR and one negative amplification control (following the single round of purification and BBN). Reproducibility of the ForenSeq mtDNA control region multiplex was assessed with three operators, each preparing libraries in quadruplicate from 100 pg, 20 and 2 pg HL60 positive control gDNA and negative amplification controls (purified water) (16 libraries total), then sequencing these on three MiSeq FGx instruments. Reproducibility of the ForenSeq mtDNA genome multiplex was assessed with three operators each preparing libraries in triplicate from 100 pg and 2 pg HL60 positive control gDNA and negative amplification controls (purified water) in duplicate using two many manufactured kits (16 libraries total), then sequencing these on three MiSeq FGx instruments.

### 2.5. Orthogonal Haplotyping for Concordance Studies

Control region libraries were prepared in triplicate at 2 pg and 100 pg gDNA using the following NIST DNA samples: SRM-2392 CHR, SRM-2392 9947A, SRM-2392I (HL60), NA03798, NA10472, the ForenSeq HL60 positive control and with four negative amplification controls (purified water). These libraries were normalized using BBN (40 libraries prepared for this study; 48 samples per run). mtGenome libraries from 100 pg gDNA were prepared in triplicate using NIST samples SRM-2392 CHR, SRM-2392 9947A, SRM-2392I (HL60), NA03798, NA10472, the ForenSeq HL60 positive control and with six replicates of negative amplification controls (purified water), normalized using BBN and split across two MiSeq FGx sequencing runs of 12 samples each.

One replicate of each control region library was prepared from 100 pg of 49 Coriell gDNAs (see Section 3.5.2), 100 pg ForenSeq HL60 positive control and one negative amplification control (purified water), normalized using BBN and sequenced. Three replicates of each mtGenome library were prepared from 100 pg of 49 Coriell gDNAs (see Section 3.5.2), 100 pg ForenSeq HL60 positive control and two negative amplification controls (purified water), normalized using BBN and split across sequencing runs with other libraries for a total of 16 libraries per MiSeq FGx run. Orthogonal mtDNA haplotype information for NIST SRM-2392 and SRM-2392I and for two Coriell DNA samples (GM03798, GM10742A) were obtained for Sanger sequencing data [52] and published next-generation sequencing data [53,54]. Orthogonal haplotype mtDNA information for the 49 Coriell DNA samples (phase 3 samples) were obtained from whole-genome sequencing data from the 1000 Genomes Project (*.vcf file available at FTP site http://ftp.1000genomes.ebi.ac.uk/vol1/ftp/release/20130502/ (accessed on 29 July 2019) [51]. Concordance was calculated based on the percentage of the same variants detected in the MiSeq FGx sequencing results relative to the rCRS for data that exceeded the default UAS analysis method for each kit, as compared to the orthogonal method(s).

### 2.6. Stability Studies

Three known PCR inhibitors for mitochondrial DNA amplification were tested using each of the ForenSeq mtDNA multiplexes. HL60 DNA at 8.33 pg/µL was incubated with calcium chloride, humic acid or *Escherichia coli* (*E. coli*) DNA at the following final concentrations: 2 mM or 3 mM calcium chloride, 70 or 100 ng/µL humic acid or 10 ng total *E. coli* DNA, for 30 min at room temperature before being added into PCR1 reactions. Concentrations of calcium chloride and humic acid were similar to those of Ewing et al. [55]. The libraries were prepared following the reference guides as described below, evaluated for average read depth (%) per base and for total known variants detected as compared to HL60 untreated DNA [56,57,58,59]. FASTQ files from samples spiked with *E. coli* DNA were uploaded to BaseSpace and analyzed using the Burrow–Wheeler aligner ((BWA) Broad Institute, Cambridge, MA, USA) application and the Kraken Metagenomics application [60,61].

### 2.7. Library Preparation

Libraries were prepared according to the ForenSeq mtDNA control region or ForenSeq mtDNA whole-genome reference guides [57,58] which use a workflow similar to that of the ForenSeq DNA signature prep kit (Verogen, San Diego, CA, USA). Briefly, samples were split across two amplifications with tagged, mitochondrial specific primer mixes (sets 1 and 2), described above, to tile across either the control region or the entire mtDNA genome (to amplify and tag targets, [57,58]) using either a GeneAmp^®^ PCR System 9700 with a gold-plated block, Veriti, ProFlex (Thermo Fisher, Waltham, MA, USA), or C1000 (Bio-Rad, Hercules, CA, USA) thermal cycler. Next, indexes were incorporated in a subsequent PCR reaction to enrich targets [57,58]), using the same index combination on the set of two amplifications for each sample. The first PCR reaction was performed in a discrete pre-PCR area, and samples were amplified using the following protocol: 98 °C initial incubation (3 min), 8 cycles of (96 °C (45 s), 80 °C (30 s), 54 °C (2 min) (with slow ramping mode dependent on the thermal cycler [57,58], 66 °C (1.5 min) (ramp at 0.2 °C per second)), 10 cycles of [96 °C (30 s) and 68 °C (3 min) (with slow ramping mode dependent on the thermal cycler [57,58], followed by a final extension at 68 °C (10 min) and an infinite hold at 10 °C. PCR2 set up and thermal cycling, for index addition (i7 and i5), were performed in a post-PCR room, [57,58], and as follows: a 98 °C initial incubation (30 s), 15 cycles of [98 °C (20 s), 66 °C (30 s), 68 °C (90 s), followed by a final extension at 68 °C (10 min) and an infinite hold at 10 °C, [57,58]. Adhesive microseals were applied to 96-well plates and sealed using a rubber roller before following steps in the ForenSeq™ protocol for shaking, vortexing, centrifugation and thermal cycling. Microseal “B” adhesive seals (Bio-Rad, part # MSB-1001) were used for shaking, centrifuging, and long-term storage (i.e., steps conducted between –40 °C to 110 °C), with suitable, skirted or semi-skirted PCR plates; Microseal “A” adhesive seals (Bio-Rad, part number MSA-5001) were used for thermal cycling. Edge wells were not used to mitigate potential evaporation.

Libraries from sets 1 and 2 were next pooled together and purified to remove primer-dimers and buffer components using sample purification beads [57,58]. Following purification, library concentrations were normalized utilizing either the bead-based protocol (BBN) or a manual quantification method (QN) by quantifying the DNA with a Quantus fluorometer (Promega Corporation, Madison, WI, USA) and adjusting the concentration of each library to 0.75 ng/µL [57,58]. The normalized libraries were pooled together (5 µL of each library) into a 1.7 mL tube for sequencing on the MiSeq FGx instrument.

### 2.8. MiSeq FGx Sequencing

To prepare for sequencing, libraries normalized using the bead-based protocol were heated for 2 min at 96 °C; 5 µL of the heated pool were immediately diluted into 600 µL of hybridization buffer (HT1) [57,58,59]. Libraries normalized using the manual quantification method were denatured with NaOH (HP3) by incubation at room temperature for 5 min and diluted with HT1. Human sequencing control (HSC) (2 μL) was denatured with NaOH (HP3) by incubation at room temperature for 5 min, then added to the pooled libraries in HT1. The HSC is a DNA library pool of 23 ForenSeq short tandem repeats (STRs) serving as a positive sequencing control for the MiSeq FGx instrument. Diluted, pooled libraries with denatured HSC in HT1 were added to MiSeq FGx sequencing cartridge (part of MiSeq FGx reagent kits, standard (for mtGenome) or micro (for control region)) and the sequencing initiated following manufacturer’s instructions [57,58,59].

Sequencing was performed on MiSeq FGx instruments with MiSeq FGx reagent kit or MiSeq FGx reagent micro kit as described in the ForenSeq mtDNA control region or whole-genome kit reference guides, and MiSeq FGx Instrument reference guide. Sequencing was performed using 151 paired-end cycles for the control region libraries or 201 paired-end cycles for the whole mtDNA genome libraries. The sequencing run also includes two eight cycle indexing reads to demultiplex the libraries utilizing the indices that were incorporated during the second PCR step. This allows a MiSeq FGx instrument to sequence and the ForenSeq UAS demultiplex data from pooled DNA libraries in a single sequencing run. Sample plexity among sequencing runs were organized as follows: eight to 48 control region samples were pooled and sequenced with a MiSeq FGx reagent micro kit, and eight to 16 mtGenome samples were multiplexed and sequenced with a MiSeq FGx reagent kit unless otherwise noted.

### 2.9. NGS Sample Multiplexing and Carryover Assessment

Supplementary Table S2E summarizes the samples, gDNA inputs, replicate numbers, purification rounds (one or two), normalization methods (BBN or QN) and reads per replicate (see Section 3.2) for sample multiplexing studies. Impacts of multiplexing and sample carryover on the MiSeq FGx were assessed in three runs (carryover study runs 1–3) for the ForenSeq mtDNA control region kit using the 47 HL60 and negative amplification control libraries described above for Sensitivity Studies. One replicate of each DNA input (10,000, 1000, 500, 200, 100, 50, 20, 10, 5, 2 or 1 pg) and a negative amplification control were pooled to generate a pool of 12 libraries (library set 1). An additional pool consisting of the second replicate of each DNA input and a negative amplification control sample was also prepared (library set 2). Immediately after running the 47-library sensitivity study (carryover study run 1), the MiSeq FGx post-run wash was conducted [57,58,59] and set 1 of 12 libraries were sequenced on the same MiSeq FGx (carryover study run 2) followed by a post-run wash. Next, library set 2 (*n* = 12) was sequenced on the same MiSeq FGx (carryover study run 3). Each of the two 12-sample sequencing runs was assessed for carryover of libraries from the previous run by using simulated samples that were assigned indexes that were not physically present in the run. This was accomplished by (1), including (via sample sheet or manual entry) 35 simulated samples and assigning to them the other 35 index combinations that were only used in carryover study run 1 (47 libraries, 47 total index combinations; See Figure S1), and (2) the 12 indexes that were present in the run. In this way, the simulated samples serve as bait for the detection of index cross-contamination during library prep and/or sequencing carryover.

### 2.10. Contamination and Crosstalk Studies

Possible contamination in NTCs for control region library preparation was estimated as average read depth per amplicon by dividing the paired depth (sample representation plot in the UAS) by 18 (the total number of control region amplicons) and multiplying by two for total depth. For the control region kit, the number of reads in the sample representation plot is similar to the paired read counts on the sample details page, such that dividing by amplicon number provides a reliable estimate for data generated with the control region kit. The total read depth divided by two estimated the paired read depth of a sample (see below *Secondary and Tertiary Data Analysis*). Possible contamination in NTCs for mtGenome library preparation was estimated in a different way because the size of the multiplex affords more opportunity for primer-dimer formation such that sample representation can inflate the number of reads relative to the paired read counts on the sample details page. For mtGenome, the “call” filter in the UAS sample details page was used to determine read depth for each position or region of interest in the mtDNA navigator. The region of coverage was determined from the position viewer. A possible future improvement to the UAS could be to filter primer-dimer from the mtDNA sample representation view.

Signal crosstalk is a term used to describe index misassignment. Crosstalk can occur if index tubes are cross-contaminated during library prep (e.g., poor pipetting technique, tube cap switch) or can occur among samples that use the same i5 or i7 index on the flow cell during sequencing. To determine if crosstalk could potentially account for reads observed in NTCs, each sample in a run that was prepared using an index that was detected in NTC(s) was compared to NTC variant call(s) using the sample Compare tool in the UAS. Read depth of the position or region detected in each NTC was estimated as in the contamination assessment described above. Under the theory that regions of highest coverage in a sample are most likely to contribute to crosstalk, the position or region with the highest read depth in the sample(s) that did not share an index with reads detected in NTC(s) was evaluated by comparing variants and indexes across the run.

### 2.11. PCR-Based Studies

PCR reaction conditions and thermal cycling parameters for the ForenSeq mtDNA control region and whole-genome kits were based on those validated for the ForenSeq DNA signature prep kit. PCR1 thermal cycling conditions were optimized for the shorter amplicons designed to tile across the control region and mtGenome as compared to ForenSeq DNA signature multiplexed amplicons. Denaturation times were tested at an increased and at a decreased 10 s increment; annealing and extension times were tested at a 30 s increased and decreased increment for the control region multiplex. Ramp rates in PCR1 were confirmed for mtGenome multiplex in increments of 2% (Veriti thermal cycler). Denaturation, annealing and extension temperatures were tested at a +2 C and a −2 C increment for both mtDNA multiplexes. The mtPCR1 and mtPCR2 buffer formulations were assessed for robustness by increasing and decreasing by 10%, 20% and 30% across critical reagents of ForenSeq mtDNA control region and whole mtGenome multiplexes to include magnesium sulfate, potassium chloride and bovine serum albumin (BSA) for mtPCR1 and mtPCR2 buffers. Both of these buffers for each kit were assessed with and without polyethylene glycol (PEG), and mtPCR1 buffer was also assessed with and without dimethyl sulfoxide (DMSO) and glycerol. These buffers were evaluated by visualizing purified libraries on the Fragment Analyzer 5300 (Agilent, Santa Clara, CA, USA) and by coverage, variant calling and amplicon balance after sequencing. The effects of multiplexing targeted amplicons were assessed by comparing variant calling and coverage of the control region between the mtDNA control region and mtGenome multiplexes. The primers that target the control region are of the same sequences and are present at the same ratios in each kit’s primer mix.

### 2.12. Species-Specificity Studies

Nonhuman DNA samples DNA were used to generate libraries to assess reads generated from the control region and mtGenome multiplexes and analyzed in the UAS. 100 pg of gDNA from two Old World primates (rhesus monkey, cynomolgus monkey), five non-primate mammals (pig, cow, dog, cat, horse), one avian species (domesticated chicken) (Zymogen Laboratories, San Diego, CA, USA), one bacterial species *Escherichia coli* (*E. coli*) (SIGMA-Aldrich, St. Louis, MO, USA) were amplified in triplicate with the control region multiplex along with triplicate positive amplification controls (100 pg HL60) and negative amplification controls (purified water) to generate 33 libraries and sequenced with other libraries for a total of 42 libraries on a MiSeq FGx using a MiSeq FGx reagent micro kit. A sample of 100 pg of gDNA each of these same 9 nonhuman samples was amplified in duplicate with the mtGenome multiplex along with duplicate positive amplification controls (100 pg HL60) and quadruplicate negative amplification controls (purified water) to generate 24 libraries and sequenced on a MiSeq FGx using a MiSeq FGx reagent micro kit.

### 2.13. Secondary and Tertiary Data Analysis

MiSeq FGx sequencing data were analyzed, and variants called using ForenSeq universal analysis software 2.0/2.1 [56]. During and after MiSeq FGx sequencing, quality metrics for the run may be viewed in run details in the UAS and may be remotely monitored during the sequencing run (these metrics mirror information displayed on the MiSeq FGx instrument during sequencing). To assist with run quality assessment, metrics are provided for cluster density, clusters passing filter, phasing, and pre-phasing. On the UAS Quality Metrics page, a color indicator displays the overall outcome of the quality metrics along with the preferred range of values for each. The “cluster density” metric (K/mm^2^) is the number of clusters (K) per square millimeter for the run. For ForenSeq mtDNA runs, a target cluster density range of 400–1650 K/mm^2^ is recommended. Cluster density values outside of the target range can still produce results that are sufficient to use for analysis. Values that deviate substantially from the target range can negatively impact other quality metrics and decrease the quantity of valuable data from the run. The “clusters passing filter” (%) metric is the percentage of clusters passing filter based on the Illumina chastity filter, which measures quality. The filter can detect low-quality base calls. The chastity of a base call is the ratio of the intensity of the greatest signal divided by the sum of the two greatest signals. If more than one base call has a chastity value of less than 0.6 in the first 25 cycles, reads do not pass the quality filter. Data for this metric are viewable after sequencing cycle 25 has been completed. For ForenSeq mtDNA samples, a cluster passing filter target value of ≥ 80% is recommended. Clusters passing filter values that are outside of this target% can still produce results that are sufficient to use for analysis. Values that deviate substantially from the target range can negatively impact other quality metrics and decrease the quantity of data from the run. The “phasing” (%) metric is the percentage of molecules in a cluster that fall behind the current cycle within read 1 and read 2 such that lower percentages are indicative of higher quality run statistics. For ForenSeq mtDNA samples, a phasing value of ≤ 0.25% is recommended. Phasing values outside of this target% can still produce results that are sufficient to use for analysis. The “pre-phasing” (%) metric is the percentage of molecules in a cluster that run ahead of the current cycle within read 1 and read 2 such that lower percentages are indicative of higher quality run statistics. For ForenSeq samples, a pre-phasing value of ≤0.15% is recommended. Pre-phasing values outside of the target% can still produce results that are sufficient to use for analysis. Each run for the validation studies passed each of these run metrics.

After MiSeq FGx sequencing, the UAS automatically demultiplexes the samples based on the supplied index sequences. Total numbers of paired reads (i.e., read 1 and read 2 for a given sample comprise one pair) for each sample in the run are counted and reported in a sample representation plot. FASTQ files are generated from the demultiplexed binary base call (BCL) files for each sample specified in the sample sheet. Reads are aligned to the rCRS using the BWA-MEM version of the Burrow–Wheeler aligner (Broad Institute, Cambridge, MA, USA) [61]. The UAS removes known nuclear mitochondrial DNA reads (NUMTs), identified by comparison to the reference human NumtS (RHNumtS) compilation as well as the human mitochondrial database (HmtDB) [62,63,64]. Mixed base positions that were not removed by the NUMT filter were also screened using BLAST analysis [65,66,67,68,69,70].

Nucleotides are trimmed based on quality such that base calls with Q scores less than 30 are removed. Unintended amplicons or byproducts (e.g., primer and/or adapter dimers), if present, of <40 nucleotides are filtered (smaller than any of the target ForenSeq amplicons, the smallest, of which is 60 bp for both ForenSeq mtDNA kits). Primers are trimmed such that the sequence of the targeted insert is reported, avoiding ambiguity in variant calling versus primers, including degenerate oligonucleotides used to increase PCR efficiency (adjacent amplicons overlap on average by 17 bp (3 bp overlap minimum) to prevent data loss due to bioinformatic trimming).

Variants are called using established forensic rules and Scientific Working Group on DNA Analysis Methods (SWGDAM) nomenclature [42]. A C-stretch describes homopolymeric runs of cytosines and is located in human mtDNA hypervariable region I (HVI) and hypervariable region II (HVII). We note that two C-stretch variants detected in these studies deviate from SWGDAM mtDNA nomenclature in the UAS: (1) 16189c (Table 1 and Section 3.5.2) in HVI should report as 16189C and 16193c as the T at position 16189 is deleted in all reads combined with a C-insertion in a portion of the reads resulting in a mixed length heteroplasmy of C’s in the C-stretch; (2) 310Y (Table 1 and Section 3.5.2) in HVII should be called 309.1, 309.2, etc., depending on the number of insertions in the read for this region. The T at position 310 is sometimes shifted during alignment, causing reads to be called 310Y. These may be corrected manually as needed until a UAS update is made.

Primer pairs that amplify the region between position 262 and position 353 achieve coverage of the C-stretch (positions 303–315) in HVII during sequencing. Sequences generated from the forward template strand have high accuracy and alignment, while those generated from the reverse template strand have high accuracy and alignment until reaching the C-stretch. Therefore, reads from the reverse strand that begin sequencing at position 262 and do not meet alignment requirements are soft-clipped after position 303 and not used for base calling. As a result, approximately half the coverage (read counts) may be obtained for positions 304–353 relative to positions 262–303 (on the reverse strand). A call is supported when it meets or exceeds the analytical threshold (AT), interpretation threshold (IT), minimum Q-score, and minimum read count. AT and IT are rounded to the maximum unobserved variant percentage to the nearest whole integer by the UAS. Unless otherwise stated in the mixture studies, default analysis settings for minimum read counts (64 or 45), analytical threshold (AT) (10% or 6%), and interpretation threshold (IT) (10% or 6%) for the ForenSeq mtDNA control region or whole-genome libraries, respectively, were used.

The default minimum read count for the “Verogen mtDNA control region analysis Method” was set in the UAS by assessing background signal on two MiSeq FGx Systems using MiSeq FGx reagent micro kits and running two sets of 48 water-only negative amplification controls across two runs. Calculating one standard deviation (30 reads) above the mean of 34 reads per position in the negative amplification controls across the 1157 positions evaluated in the control region provided the default minimum read count value of 64 reads per base at a position. The same approach was used for the “Verogen mtDNA whole-genome analysis method”. Two MiSeq FGx runs, using the MiSeq FGx reagent kit, of 16 water-only negative amplification controls (NTC), indicated three standard deviations (42 reads) above the mean of 1 read per amplicon in the negative amplification controls, across the 245 amplicons that cover the entire mtDNA genome, yielding default minimum read count value of 45 reads per base at a position.

The default analytical threshold (AT) for the “Verogen mtDNA control region analysis method” was set by assessing background signal across four operators on four MiSeq FGx instruments using MiSeq FGx reagent micro kits. Libraries were generated using the ForenSeq mtDNA control region kit and sequenced in 10 runs. Each run sequenced two to 18 ForenSeq mtDNA positive control DNA libraries (HL-60) for a total of 63 positive amplification controls at 100 pg each. After sequencing, data were analyzed in ForenSeq UAS v2.0/2.1 using a minimum read count of 64 and AT and interpretation threshold (IT), each set to 0%. The average percentage of unexpected variants across the ten runs was 0.7% (1% standard deviation), with a range of 0.1% to 9.7% (4157 bases out of 72,954). The 95th percentile for unexpected variants was 2.2%. The range for maximum percentages of unexpected variants was 3.9% to 9.7%. Each maximum data point occurred within the hypervariable I (HVI), and hypervariable II (HVII) C-stretches or the AC repeat at positions 523–524. Excluding these locations, the maximum percentage of unexpected variants was 2.1%. These data provided a default 10% AT and IT for the Verogen mtDNA control region analysis method.

Similar assessments were conducted for the “Verogen mtDNA whole-genome analysis Method” across six operators on five MiSeq FGx instruments using MiSeq FGx reagent kits. Libraries were generated using the ForenSeq mtDNA whole-genome kit and sequenced in six runs. Each run sequenced two to 16 ForenSeq mtDNA positive control DNA libraries (HL-60) for a total of 90 positive amplification controls at 100 pg each. After sequencing, data were analyzed in ForenSeq UAS v2.1 using a minimum read count of 45 and AT and IT, each set to 0%. The average percentage of unexpected variants across the six runs in the 90 HL-60 positive control samples of 0.7% (0.5% standard deviation), with a range of 0.1% to 5.3% (16,203 bases out of 1,491,210). The 95th percentile for unexpected variants was 1.6%. The range for maximum percentages of unexpected variants was 1.9% to 5.3%. These data provided a default 6% AT and IT for the Verogen mtDNA whole-genome analysis method.

Read depth is shown on the samples details page in the UAS, for each base in either the control region or the mtGenome depending on the library preparation kit used. The total read depth for each base is displayed in the position viewer and graphically in the coverage plot. The UAS assists in viewing stranded-ness by displaying strand depth as the read count number produced from the strand (forward or reverse) that has the majority of reads for a nucleotide position. The read count for the opposite strand is then the remainder of the displayed Total read count (total number of reads, from both strands for an mtDNA base) minus the displayed strand depth. Variant call format (VCF) and binary alignment map (BAM) files are conveniently available on the UAS Server at a specified, “clickable” path for convenient access if one wishes to explore additional bioinformatic tools, such as the integrated genomics viewer (IGV; Broad Institute, Cambridge, MA, USA), especially for forensic research studies and database QC efforts.

To determine the haplogroups, reports were generated in the UAS that are compatible with the European DNA Profiling Group (EDNAP) mtDNA population (EMPOP) database (“EMPOP reports” (.txt file)) were generated in the UAS for individual samples or groups of samples. These reports contained two formats either, of which is EMPOP-compatible (FASTA format of the sample’s sequence string and a list of variants in the rCRS format) was opened with a text editor and data were queried on the EMPOP website at https://empop.online/ (accessed on 19 October 2020) to determine the haplotype of a given sample [71,72].

## 3. Results

### 3.1. Mock Casework Samples, CR Concordance between Multiplexes

#### 3.1.1. Dental and Bone Samples

Mock casework samples from five dental remains (InnoGenomics) and from 11 bones were analyzed using the ForenSeq mtDNA control region and ForenSeq mtDNA whole-genome multiplexes (Table 1) and sequenced at a plexity of 48 samples, and 10 samples total per MiSeq FGx run, respectively. Bone DNA extracts included seven contemporary samples from SHSU’s AARC Outdoor Research Facility exposed to various environmental insults, including commercial cremation (from funeral home), burning, embalming, and partial decomposition with internment, and four ancient, Eastern European, interred bone samples from PSU. The 16 challenged samples were processed with the ForenSeq mtDNA control region kit (normalized using QN) and the ForenSeq mtDNA whole-genome kit (two rounds of purification followed by QN). Complete control region coverage was observed for all 32 ForenSeq libraries between the two multiplexes (Table 1).

**Figure 2:**
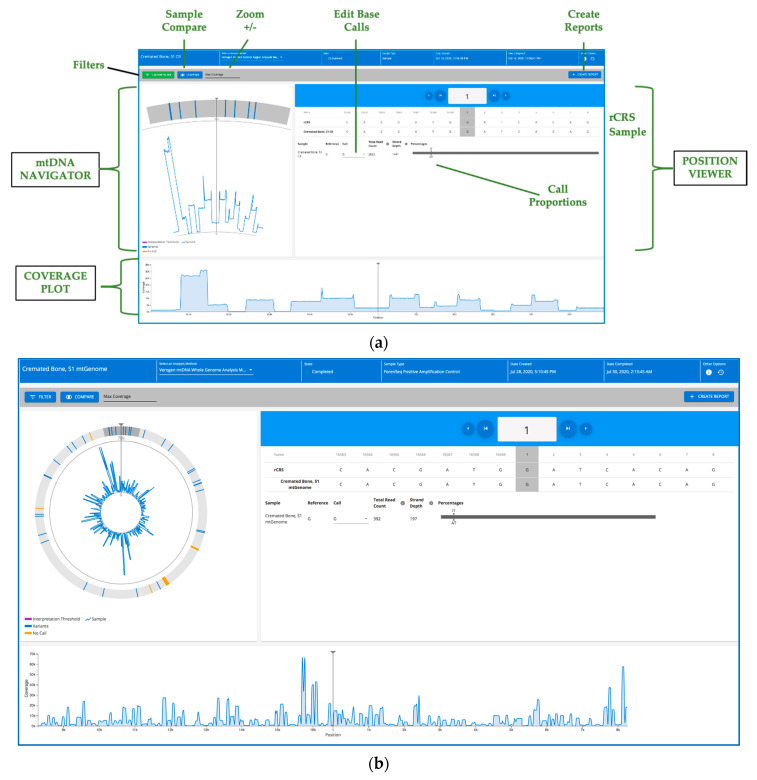
mtGenome data visualization of a mock casework sample as displayed in ForenSeq universal analysis software (UAS). (**a**) “Sample details” view zoomed to control region only; (**b**) zoomed out to display the whole mtGenome. The three main sections of the UAS sample details view are labeled in (**a**) and shown in (**b**): mtDNA navigator (upper left), Position Viewer (upper right) and coverage plot (bottom); a subset of software options and tools are also labeled in (**a**). (**a**) Commercially cremated bone sample S1 view of control region with 100% coverage and 100% variant calls reported. Variants are indicated by blue-colored tick marks in the mtDNA navigator; zero orange-colored tick marks are displayed, indicating complete coverage and zero “no calls”, which would render in orange (see (**b**)). (**b**) Bone sample S1 zoomed out view of entire mtGenome, as compared to (**a**) with 98% coverage due to “no calls” in eight regions (see Table 1), which are visible as orange-colored tick marks in the mtDNA navigator circle. Notes: 100% control region variant concordance was observed between both kits for bone sample S1; base call “C” was present at position 16,183 at 6.3% (below default AT) in control region run and at 6.3% (above default AT) in mtGenome run (Table 1).

Coverage of the mtGenome ranged from 96% to 100%; when a loss of coverage (positions where read numbers did not exceed the default minimum read count and AT) occurred, it was outside of the control region (Table 1 column “mtGenome no call regions”; Figure 2) and ranged from one position to 17 amplicons (highest data loss by amplicon count was in embalmed bone sample S2). Variants outside of the control region in the mtGenome sequencing results are shown in Supplemental Table S3. Of the 3920 mtGenome targeted amplicons in this study, five amplicons (0.13%) dropped out due to primer-binding site mutation in the following samples and regions: tooth 1662 (8290*–*8379), bone S2 (11187*–*11189), bone S3 (7216*–*7310), bone S5 (12466–12536 and/or 12563–12614) and bone P73 (15520–15581). Each of these was not part of the ForenSeq design as they occur at <1% (ranged from 0.1%–0.35%) in the MitoMap sequence dataset [46].

Discordant control region variant calling was not observed between the two kits (Table 1, columns “CR Observed Variants” and “mtGenome CR Observed Variants”), which employ the same control region primer sets, for reads that exceeded the default analysis parameters (Table 1 footnotes), with two noted observations: for Tooth sample 1661, mixed bases were detected at one position (497 M) in the control region of one multiplex, likely as drop-in or error; for bone sample P73, 10 mixed base positions were detected in the control region of the mtGenome that were either not detected (zero reads) or fewer than the AT in the control region multiplex (Table 1 footnotes) as follows: 310Y, 557Y and eight “Y” calls between positions 459 and 518. In addition to P73 with 10 mixed base variants, we note that in the other three ancient, interred bone samples, the number of mixed base variants detected ranged from one (P43pt1) to six (P2, P48) whereas the 12 contemporary samples carried none or one. In investigating further these mixed bases in bone samples P2, P48, P73 and associated blanks could not identify a specific source of possible contamination from other samples in the library preparation, nor as a potential NUMT (see Materials and Methods). Call differences here are likely due to differing read depths between the control region and mtGenome sequencing runs. In this mock casework study, control region samples were sequenced at 48 sample plexity, whereas 10 mtGenome samples were batched per run. Most of the differing calls are due to fewer reads than the AT in control region kit data. When analyzing samples of varied DNA inputs and/or quality, detection of both calls at sites of true heteroplasmy may be hampered as read numbers may be fewer than the default AT/IT or minimum read count in the ForenSeq UAS for these types of challenging samples. When the investigation of indels, heteroplasmy/mixed length, or mixed point variants are of interest, it can be prudent to consider fewer samples per run or application of a custom analysis method (lower threshold(s)) in the UAS for indels based upon internal validation. Sanger sequencing was attempted on bone samples S1–S7. The quality of the sequencing results was most likely impacted by PCR inhibitors in the samples resulting in a high percentage of mixed bases, making concordance comparisons difficult. These bone and tooth samples were also processed with the PowerSeq™ CRM nested system, sequenced on the MiSeq FGx and analyzed using the Verogen mtDNA control region analysis method. Concordant variant calling with those shown in Table 1 was observed, with the exception of a PCR error or drop-in of a C at position 545 (545S) in all samples in Table 1 (including the HL60 positive control) except for samples 1661 and 1665.

EMPOP-derived haplogroups assignments, based on control region variants only (left side of Table 1) and on whole mtGenome variants (right side of Table 1), highlight information refinement that can be gained when the entire molecule is considered. When appropriate, these more comprehensive data may better inform research interests or actionable investigative lead generation in forensic or humanitarian matters.

#### 3.1.2. Buccal Samples and Rootless Hair Shafts

Matched sets of three samples from six individuals were prepared as a buccal swab and two rootless hair shaft samples of 0.5 cm and 2 cm in length (individuals 2, 4, 5, 8, 11, 12; Table 2). Performance of the ForenSeq mtDNA control region and whole-genome kits and ForenSeq software was assessed with 36 libraries in three MiSeq FGx sequencing runs. ForenSeq control region and mtGenome libraries were generated from 100 pg gDNA from buccal samples or 12 uL hair extract. The 18 control region and 18 mtGenome libraries were sequenced at a plexity of 48 and 16 samples per run, respectively. Data generated with the control region multiplex produced complete coverage for 11 of 18 samples, with some no calls observed in the HVII C-stretch for seven samples (Table 2). No primer-binding site variants were detected in the control regions among these mock casework samples. When reads that exceeded the default analysis parameters from the 2 cm and 0.5 cm hair extracts were compared to buccal sample data for the six individuals tested, all variants were concordant within control region multiplex data (Table 2).

Complete control region coverage was observed for all 18 mtGenome samples. When comparing samples across kits, control region variants with reads that exceeded the default analysis parameters were concordant with observations of possible heteroplasmy (positions 309, 315) as footnoted in Table 2, and the presence of 489Y in one sample of the 6 samples tested from individual 12 that is not traceable to a contamination event. Complete mtGenome coverage was observed for 12 of 18 samples, with some no calls observed in six samples (Table 2). Of the 4734 targeted amplicons in this study, one (0.02%) did not exceed default analysis parameters (positions 9489–9526) due to a mtGenome primer-binding site mutation in individual 11′s 0.5 cm hair sample (reported at <1% frequency [46] so not part of ForenSeq design). Of the positions covered, whole mtGenome data were 100% concordant for the 2 cm and 0.5 cm hair extracts as compared to buccal samples (Table 2, Supplemental Table S4). For these studies, the maximum recommended sample plexities for 100 pg high-quality DNAs were run for each multiplex; in some scenarios and depending upon project or case-specific goals, it may be advantageous to consider fewer libraries per run in order to increase read depth (e.g., when handling low inputs or partially degraded mtDNA). EMPOP-assigned haplogroups for four of the six individuals were refined when using comprehensive variation across the mtGenome rather than the control region alone (Table 2).

### 3.2. Sensitivity Studies

Sensitivity studies of target gDNA input, effects on variant call rates across a range of DNA inputs and limit of detection for each mtDNA kit, under specified parameters, including sample multiplexing in MiSeq FGx sequencing and quality of libraries pooled in the sequencing reaction regarding purification and normalization were conducted.

#### 3.2.1. Control Region Multiplex: Dilution Series, Library Purification & Library Normalization, Sample Plexity 

**Figure 3:**
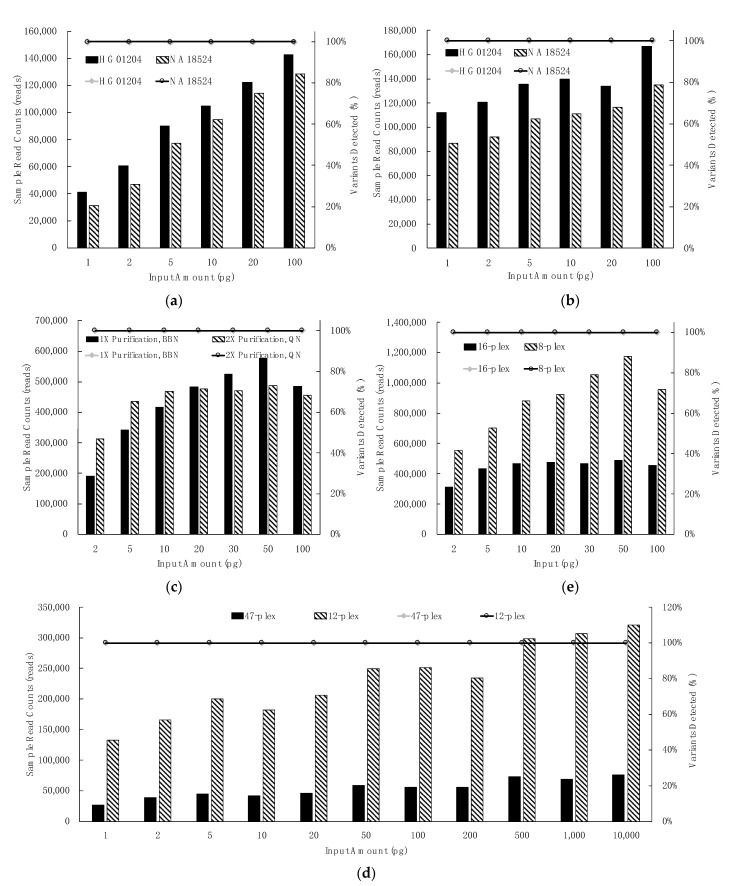
Sensitivity studies of the control region and mtGenome multiplexes: DNA inputs, library purification and normalization methods, sample plexity. Various gDNA template inputs (*x*-axis) relative to total reads per sample (*y*-axis, left), and relative to detection of expected variants detected under a set of condition(s) (*y*-axis, right) plotted as open and closed circles shown in horizontal one atop each graph. (**a**) control region multiplex: dilution series of gDNAs HG01204 (solid bars, closed circle) and NA18524 (hatched bars, open circle). Libraries were prepared in duplicate, purified 1× and normalized with BBN (average of two reps plotted), (**b**) control region multiplex: dilution series of gDNAs HG01204 (solid bars, closed circle) and NA18524 (hatched bars, open circle). Libraries were prepared in duplicate, purified 2× and normalized with QN (average of two reps plotted), (**c**) mtGenome multiplex: dilution series of HL60 gDNA. Libraries were prepared in duplicate, purified 1×, normalized with BBN (solid bars, closed circle), or purified 2× and normalized with QN (hatched bars, open circle) (average of two reps plotted), (**d**) control region multiplex: dilution series of HL60 gDNA. DNA libraries were prepared in quadruplicate with three negative amplification controls, purified 1x and normalized with QN. MiSeq FGx runs using micro sequencing kit were conducted with either 12 or 47 sample plexity, shown as solid or hatched bars, respectively, (**e**) mtGenome multiplex: dilution series of HL60 gDNA. Libraries were prepared in duplicate, purified 2× and normalized with QN. MiSeq FGx runs using standard sequencing kit were conducted with either 8 or 16 sample plexity, shown as solid or hatched bars, respectively.

Sensitivity studies using the ForenSeq mtDNA control region kit with MiSeq FGx reagent micro kit were performed with three high-quality DNAs: NA18524 and HG01204 (each in duplicate) and HL60 (in quadruplicate). Sensitivity study libraries generated with NA18524 and HG01204 (1, 2, 5, 10, 20, and 100 pg total gDNA input) and one round of purification were normalized using the two methods recommended for this kit: bead-based normalization (BBN, Figure 3a) and manual quantification and normalization (QN, Figure 3b). All expected variants for NA18524 (eight single nucleotide variants (SNVs), one insertion, one deletion) and HG01204 (eight SNVs, one insertion, five deletions) were detected as greater than the default AT for all input amounts and both normalization methods (Figure 3a,b, circles/horizontal line atop graph). Increased reads were obtained for inputs less than 20 pg gDNA when the QN method was used such that it may be prudent to consider QN instead of BBN to maximize read depth when samples less than 20 pg are to be analyzed.

#### 3.2.2. mtGenome Multiplex: Dilution Series, Library Purification and Normalization, Sample Plexity

Sensitivity studies for the ForenSeq mtDNA whole-genome kit with MiSeq FGx reagent kit were performed with high-quality HL60 gDNA (2, 5, 10, 20, 30, 50, 100 pg), once with one round of bead purification followed by BBN (Figure 3c, solid bars) and once using two rounds of bead purification followed by the QN method (Figure 3c, hashed bars). Data indicate that two rounds of bead purification coupled with the QN method can enhance data recovery from the mtGenome for samples at less than or equal to 20 pg gDNA input. This second optional purification entails ~15 m of hands-on time and may assist with damaged or degraded samples as well as detection of low-level heteroplasmy (Figure 3). All expected variants for HL60 DNA were observed at every input (34 SNVs, one insertion, one deletion) (Figure 3c, circles/horizontal line atop graph).

#### 3.2.3. Extent of Sample Multiplexing in Sequencing: Depth of Coverage (DoC)

The maximum number of mtDNA libraries to sequence simultaneously using a MiSeq FGx reagent kit (standard or micro) depends upon variables, such as the total number of targeted nucleotides and the desired number of reads per mtDNA nucleotide per sample (read depth). Complete coverage was generated, using default UAS settings, from a maximum of 48 control regions (micro runs, 100 pg HL60) and of 16 mtGenome (standard runs, 100 pg HL60). For example, three sequencing runs for the control region multiplex, and two for the mtGenome were assessed for effects of two sample multiplex levels on read depth (Figure 3d,e). HL60 gDNA was tested in quadruplicate (from 1 pg to 10 ng total input, BBN); 47 control region libraries were sequenced in a micro run, then libraries were subsequently re-pooled and re-sequenced twice at the 12-plexity level. The expected ~four-fold deeper coverage (increase in 12-plex runs ranged from 4.1 to 4.6x) was generated in the 12-plex runs (2.55 M average total reads) as compared to the 47-plex (587 K total reads). All expected variants were detected at all input levels (eleven SNVs and one insertion) in a 12 sample and 47 sample run (Figure 3d, circles/horizontal line atop graph). Similarly, for the whole-genome multiplex, HL60 gDNA was tested in duplicate (from 2 to 100 pg total input, QN); libraries were run at a 16-sample multiplex level followed by re-sequencing at an eight-sample plexity. The eight-plex run (6.25 M total reads) generated the expected approximate doubling of total reads as compared to the 16-plex (3.19 M total reads; Figure 3e). All expected variants were detected at all input levels at eight and 16 sample plexity per run (Figure 3e circles/horizontal line atop graph).

Predictable total read count trends may be obtained when varying the number of samples per run. Deeper coverage observed in the 12-plex control region and in the 8-plex mtGenome data did not adversely impact variant calling (Figure 3). Running fewer than the maximum number(s) described here may be considered analogous to increased injection time on a capillary electrophoresis genetic analyzer. A minimum of eight mtDNA control region or mtGenome libraries is recommended to be processed at a time, including positive and negative controls if used, to avoid the introduction of pipetting inaccuracies when preparing master mixes due to small volumes. Because of the exceedingly high number of reads that may be produced at this lower plexity, the use of unique index combinations for eight sample runs (the supported minimum) may be helpful if this very deep sequencing is needed for some reason. Ultimately, the range of sample numbers multiplexed would be determined by the operational forensic lab and may consider the quantity and quality of the libraries to be run simultaneously on a flow cell.

### 3.3. Mixture Studies

ForenSeq libraries were generated for two-person mixture samples of gDNA template at two total inputs (5 pg, 100 pg). Table 3 summarizes ratios of 2800 M (minor) DNA to HL60 (major) DNA at 1:3, 1:5 and 1:15 for the control region kit analyzed with a custom 3.7% AT/IT. Single source 2800 M carries 10 variants relative to the rCRS in the control region; each minor variant was detected above 3.7% AT/IT, except at the 5 pg input of 1:15 ratio where 20% of minor donor variants either dropped below 3.7% AT or were fewer than the minimum read count (64 reads). As noted in Table 3, one unexpected variant (501Y), unknown to 2800 M and HL60, was detected in one control region 5 pg library and not traceable to a contamination event. Using this custom 3.7% AT (0.7% +3 SD; see Materials and Methods) in the UAS with control region multiplex data, 3.5% of minor contributor variants were called (147 of 4157) in C-stretches (~40% 310Y, ~30% 524c, ~20% 315.c) and AC repeat (~10% 523a). An AT lower than the default, such as 3.7% AT used here, could be considered if, for example, it is desired to detect less than 10% mixed bases, heteroplasmy or minor contributor(s) in mixtures. For example, with careful interpretation of the HVI and HVII C-stretches and AC repeat (~523,524), a custom 3.7% AT may assist with detection and interpretation of heteroplasmy or minor contributors at 5%. Increased read numbers produced by, including fewer samples per run, can assist in mixture analyses instead of or in addition to the custom threshold(s).

A similar summary is provided in Table 3 of mtGenome data for two-person mixture samples of gDNA at two template inputs (5 pg, 100 pg) at ratios of 2800M (minor) to HL60 (major) at 1:1, 1:3 and 1:9 using default analysis parameters. Single source 2800M carries 27 variants across the entire mtDNA molecule; each of these minor variants was detected above the default 6% AT/IT, except at 1:9 ratios where 15 and 17 of expected minor variants were detected at 100 pg and 5 pg, respectively as reads were less than default AT or default minimum read count of 45 reads for mtGenome. When a custom 3% AT (0.7% +3SD, rounded up; see Materials and Methods) was applied, 0.6% of minor contributor variants were called (91 of 16,203) in C-stretches, AC repeat, and other homopolymeric regions (e.g., poly-A tract at 12,418–12,425). Lowering the AT could be considered if, for example, it is desired to detect less than 6% mixed bases, heteroplasmy or minor contributor(s) in mixtures. As with the control region multiplex, with careful interpretation of the HVI and HVII C-stretches, AC repeats at positions 523–524, and other homopolymeric regions, a custom 3% AT may assist with detection and interpretation of heteroplasmy or minor contributors at 5%. As stated, to increase read numbers, fewer samples per run is a consideration as well.

Filtering the view in the UAS to display variants can assist when delving into a possible mixture. Mixtures were visually indicated in the UAS as numbers of variants and of mixed bases were observed to be increased relative to single-source samples. Single source mtDNA molecules carrying single base changes or insertions and deletions with either no or a low percentage of heteroplasmic sites may be distinguished between data from mixed DNA samples where >50% mixed-base variants may be observed, as in this mixture study.

### 3.4. Reproducibility and Repeatability: Precision, Accuracy (Concordance), Average Coverage

Repeatability and reproducibility studies were conducted as described in Materials and Methods and included multiple operators, multiple MiSeq FGx instruments and 12 runs. Data indicate precise and repeated variant calling for the control region and the mtGenome among gDNAs, among libraries, among MiSeq FGx, runs and instruments that were reproducible among operators. Precision was calculated by determining the bases covered as a fraction of the total bases expected for either the control region or mtGenome across MiSeq FGx runs. Data indicated 100% precision in control region variant calling (HL60, 2 pg and 20 pg) and ranged for mtGenome from 97.9–99.98% (nine DNAs, 2 pg, 20 pg, 100 pg) due to incomplete coverage (see Table 4 footnotes) in a subset of samples ranging on average from four nucleotides (five Coriell DNAs) to 356 nucleotides (HL60 2 pg). Precision in variant calling was examined by calculating the maximum difference in variant frequencies for each variant in each sample between and within runs [26]. HL60 has no mixed base heteroplasmy in the control region; all variants were detected at 100% frequency with a maximum difference of 0%. Length and mixed length heteroplasmy frequencies varied between samples and runs with a maximum difference observed of 5% for repeatability runs and 8% for reproducibility runs. Both mixed base and length heteroplasmies were observed in the mtGenome runs. All base substitutions were observed at 100% with a maximum difference of 0%. The maximum difference in frequency of mixed bases was 9.6% for the repeatability runs, and 18.2% for the reproducibility runs. The maximum difference in frequencies for length and mixed length regions observed was 3.3% for repeatability runs, and 24.1% for reproducibility runs. Automated ForenSeq variant calls for data produced with the control region multiplex, and the mtGenome multiplex using gDNA samples ForenSeq positive control HL60, five Coriell samples HG02190, HG02215, HG02449, HG01497, NA12870 and NIST SRM 2392-I (HL60, 9947A, CHR) were considered accurate and concordant when they were the same calls as those produced with orthogonal sequencing methods (i.e., Sanger sequencing using capillary electrophoresis or whole-genome sequencing (WGS) using NGS/MPS). Concordance of ForenSeq variant calling of the control region and the mtGenome, relative to Sanger-type and whole-genome sequencing, was repeatable and reproducible at 100%, with point heteroplasmy at positions 1490 and/or 4821 not detected in some mtGenome replicates (see Table 4 footnotes).

### 3.5. Additional Concordance Studies

#### 3.5.1. Control Region Concordance Between ForenSeq Multiplexes

Concordance studies of variant calling in the control region, relative to the rCRS, were conducted by comparison between the two ForenSeq mtDNA kits using five known DNA samples and default UAS analysis settings (Table 5). Control region haplotypes for these samples generated with ForenSeq multiplexes were the same as previously reported and were the same between the two multiplexes [52]. This included the expected heteroplasmic point and length differences in sample CHR between Sanger-derived and NGS-derived haplotypes [53,54]. In this study, heteroplasmy in CHR was observed at position 64, as was the 16193.1c insertion, each of which was undetected in Sanger sequencing. Data indicate complete control region coverage and concordant variant calling in the control region and the mtGenome for the five well-known samples tested at 100 pg total gDNA inputs (Table 5, Supplementary Table S5; 48 and 12 samples per run for control region multiplex and mtGenome, respectively) [52,53,54].

#### 3.5.2. Concordance and Orthogonal Methods, Haplogroup Assignments, Population Studies

Concordance studies of variant calling, relative to the rCRS, in the control region and of the mtGenome were conducted using 49 gDNA samples from Coriell (100 pg, Table 6) as described in Materials and Methods. Comparisons were made among control region data produced from each ForenSeq kit and the 49 Coriell samples as well as between the ForenSeq full mtGenome data and publicly available 1000 Genomes Project data for these Coriell samples. Complete coverage and concordant control region typing was observed in each sample for each multiplex with one exception where sample HG00844 was missing 49 bases (positions 470–519) in the mtGenome data (Table 6, far-right column, “*mtGenome Multiplex: No Call Region(s)*” column; 48 and 16 samples per run for the control region and mtGenome multiplexes, respectively). We observed the following: (1) 189R called in mtGenome multiplex data from HG01205 present at 1226 reads in control region multiplex data at 9% just under the 10% default AT used in this study; (2) in 11 samples, a total of 16 instances were observed where reads in the control region multiplex at a hotspot (C-stretches, *n* = 13, for example, 309.1C and 309.1c, and/or AC-repeat, *n* = 3) were fewer than the default AT (Table 6, samples HG00844, HG00384, HG01197, HG01790, HG02190, HG02238, HG02239, HG02322, HG02513, NA12874, NA20509).

Of the covered mtGenome bases, 100% concordance was observed as compared to 1000 Genomes Project sequencing data (Supplementary Table S6). Incomplete mtGenome coverage outside of the control region was observed in three of the 49 samples; four regions that ranged from 42 to 67 bases and one 131 bp region were not called in samples HG00844 and HG02322, respectively, due to reads < default AT% (Table 6, 16 samples per run). Loss of coverage of positions 6922–6988 and of 7256–7365 in samples HG00181 and HG02322, respectively, were attributed to rare variants occurring in the MitoMap dataset (24 May 2019 update) at <1% [46]. Refined haplogroup assignment was enjoyed by 60% of these 49 samples when using EMPOP and the entire mtGenome as compared to control region variants only.

### 3.6. Population Analyses and Studies

mtGenome sequences among populations and lineage distributions, as reported in MitoMap, helped to form degenerate oligonucleotide designs for PCR primers [46]. Variants reported at >1% frequency, among 48,882 mtGenome sequences, were included as wobble bases to promote successful PCR extension among phylogenetically distinct samples when using ForenSeq kits. At least eight and as many as 9167 mtGenome sequences were considered among 33 macro-haplogroups and subclades (Table 7).

The 71 samples reported in Table 1, Table 2 and Table 6 represented four major population groups, 15 macro-haplogroups (B, C, D, F, H, J, K, L, M, N, R, T, U, W, X) and 63 unique EMPOP-derived haplogroups and served as a small population study (Refer to Supplementary Table S1 for additional Coriell expanded ancestries) that challenged the ForenSeq kits. As reported in Section 3.1.1, Section 3.1.2 and Section 3.5.2, after investigating mtGenome regions of “no call”, seven primer-binding site mutations were observed (0.04% of 17,395 mtGenome amplicons) that reduced PCR efficiency to the point that reads were not detected or were fewer than required for the instant analysis parameters. These rare variants (<1% frequency) were not part of the 237 degenerate oligonucleotides in the mtGenome multiplex. Control region primer site variants were not detected.

### 3.7. Contamination Assessment

#### 3.7.1. Exogenous DNA

Potential exogenous DNA in libraries that aligned to human mtDNA (e.g., sample-to-sample contamination during library preparation) was assessed across 28 MiSeq FGx runs (13 control region, 15 mtGenome) in 74 negative template controls ((NTCs), 44 and 30 for the control region and mtGenome libraries, respectively) from studies of sensitivity, mock casework, mixture, repeatability and reproducibility for each multiplex and one species study run for mtGenome. NTC data were viewed in the UAS using default analysis parameters for each kit and evaluated. On average, 26 bases were covered among the 44 NTCs prepared along with control region multiplex libraries, with 35 of these NTCs having no reads and nine with one region (*n* = 5), two regions (*n* = 2), four regions (*n* = 1) or five regions (*n* = 1) detected that ranged from 57 to 317 total bases. The average reads in the covered region(s) across these nine NTCs ranged from 65 reads (1 region of 57 bases) to 450 reads (1 region of 68 bases). On average, 100 bases were covered among the 30 NTCs prepared along with mtGenome multiplex libraries, with 16 of these NTCs having no reads and 14 with one region (*n* = 6), two regions (*n* = 2), three regions (*n* = 3) and *n* = 1 each for four, five or six regions detected that ranged from 58 to 508 total bases. The average reads in the covered region(s) across these 14 NTCs ranged from 50 reads (1 region of 58 bases) to 310 reads (4 regions of 508 bases).

When investigating the potential source of reads among the 23 NTCs with reads, sample-to-sample contamination within each study was not detected with the following two possible exceptions in mtGenome data: one NTC (reproducibility study) contained a total of one region and one variant (73G, 50 reads) that is also in the HL60 control DNA; one NTC (species study) contained a total of one region and one variant (750G, 50 reads) that is also in HL60. Data were compared among the 19 NTCs that were prepared as part of replicates (11 sets for control region: eight sets for mtGenome): duplicates (*n* = 4), triplicates (*n* = 2), quadruplicates (*n* = 12), sextuplicate (*n* = 1)). Of these, each NTCs had zero reads in the UAS in three NTC sets; more than one NTC had reads in seven sets, allowing for comparison: one example was observed of a control region quadruplicate where two samples had coverage of an overlapping region (16,127–16,197 and 16,129–16,197) with 90 and 155 average reads.

#### 3.7.2. Signal Crosstalk

The possibility of signal crosstalk on a flow cell within MiSeq FGx runs was assessed among the 74 negative template controls (NTCs) described in Section 3.7.1. No instances of crosstalk were observed among samples on the same run and reads in NTCs that had an i5 or an i7 indexed adapter in common and shared the same variants within the region(s) of coverage.

#### 3.7.3. Sample Carryover between Runs

Potential for carryover of DNA from libraries previously sequenced on the MiSeq FGx was assessed among three runs that were conducted within the sensitivity studies reported herein that used 12 DNA samples, prepared in quadruplicate, and referred here as library sets. Following the sensitivity sequencing run of 48 control region libraries (carryover study run 1), the 12 libraries that comprise library set 1 were pooled independently and sequenced on the same sequencer (carryover study run 2) after the prescribed post-run wash procedure was conducted [59]. Carryover study run 2 included all 48 index combinations that had just been run in the carryover study run 1 (48 sample plexity). Subsequent to carryover study run 2 and the post-run wash cycle, the 12 libraries that comprise library set 2 were pooled independently and sequenced on the same sequencer (carryover study run 3) and included all 48 index combinations again. In carryover study runs 2 and 3 (each of the two 12-plex runs), no reads were detected for the 36 index combinations from carryover study run 1 (the indexes that were not physically included in runs 2 or 3). Reads were detected only for each of the 12 index combinations used for carryover study runs 2 and 3 with 12 independent pooled libraries each. Run-to-run carryover was not detected in the remainder of our studies. It is important to conduct the MiSeq FGx post-run wash and the weekly maintenance wash [59].

### 3.8. PCR-Based Studies

#### 3.8.1. Reaction Conditions

Reaction conditions were determined that provide robust forensic performance windows and included thermal cycling parameters and PCR components. Thermal cycling conditions were optimized for multiplexed PCR efficiency and specificity, reduced primer dimer formation, and balance among amplicons. The recommended and validated thermal cycling conditions provided at least a +/−10 s window for denaturation, a +/−30 s window for annealing and extension and a +/−2% ramp rate window for both mtDNA multiplexes as indicated by data from incremental time and temperature testing.

The mtPCR1 and mtPCR2 buffers were assessed for robustness and limitations by increasing and decreasing by 10%, 20% and 30% reagents, one at a time, in the ForenSeq mtDNA control region and whole mtGenome multiplexes. Tested components included magnesium sulfate (MgSO_4_), potassium chloride (KCl) and bovine serum albumin (BSA) for mtPCR1 and mtPCR2 buffers. mtPCR1 and mtPCR2 buffers for each kit were also assessed with and without polyethylene glycol (PEG), as well as mtPCR1 buffer with and without dimethyl sulfoxide (DMSO) and glycerol.

**Figure 4:**
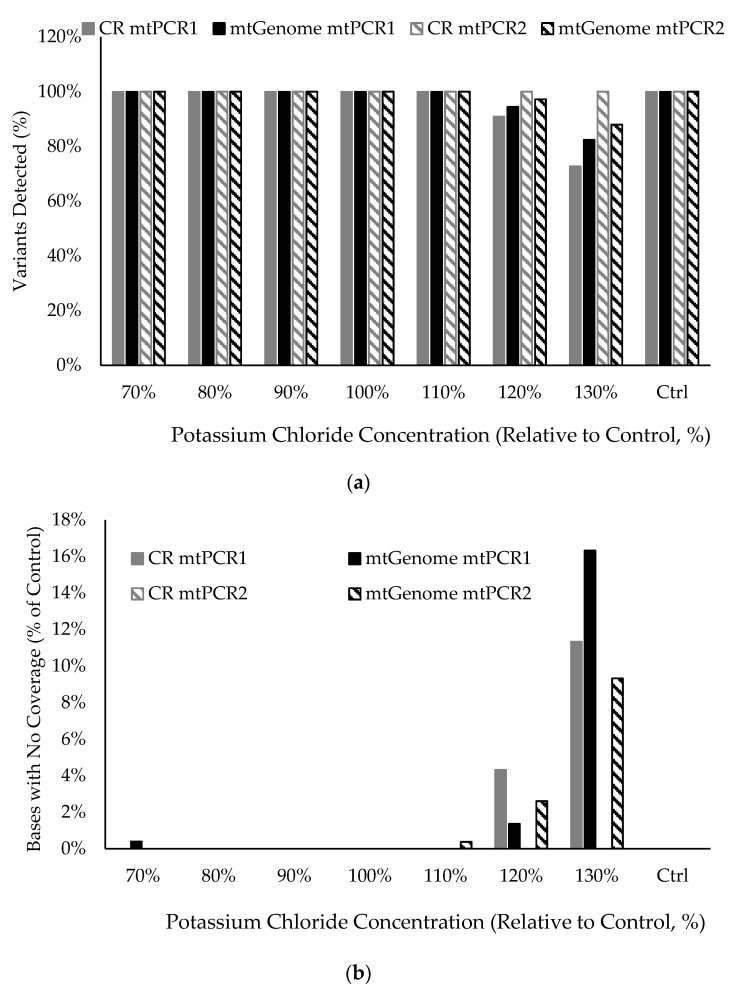
Critical reagents in PCR-based studies: potassium chloride concentration effect on variant detection and coverage in the control region and mtGenome multiplexes. Effects of varied KCl concentrations on the four mtDNA PCR buffers (mtPCR1 and mtPCR2 for each kit; x-axes in (**a**) and (**b**)) on variants detected (% relative to total; *y*-axis in (**a**)) and on bases with no coverage (% relative to control; *y*-axis in (**b**)) was assessed using 100 pg of HL60 positive control gDNA. 100% KCl is the titration control, and “Ctrl” is the commercial buffer lot of mtPCR1 and mtPCR2 for both multiplexes. Increased KCl relative to the control, and thus the manufactured standard, can contribute to data loss, as has been reported for forensic PCR systems generally.

Specifically, components of mtPCR1 and/or mtPCR2 buffers, tested in dilution series during development included MgSO_4_, KCl, and BSA at −30%, −20%, −10%, +0%, +10%, +20%, +30% where +0% is the final manufactured concentration. Except for KCl, library concentration and average size as determined on Fragment Analyzer 5300 (Agilent, Santa Clara, CA) were not affected for libraries prepared across these % windows or +/−PEG, DMSO and glycerol, nor were variant call rates, coverage of bases, and balance of amplicons (Figure S3). KCl is a critical reagent in mtPCR1 and mtPCR2 buffers for both kits, as shown in Figure 4. Reduction of KCl to 70% of the control concentration of mtPCR1 or mtPCR2 buffer of each multiplex had no effect on variant call rates but caused <2% loss of base calls at 100 pg gDNA input when reduced in the mtPCR1 buffer of the mtGenome kit due to amplicon imbalance. Conversely, data indicate that when this salt is increased above the control concentration by 120% or more for three of the four mtPCR buffers, then variant detection decreased from 100% to ~70–90%, except for in the mtPCR2 control region kit buffer, which was more resilient in this test and maintained 100% variant detection out to the tested extreme (130%). When KCl is increased to 110%, 120% and 130% of control concentration in mtGenome mtPCR2 buffer, data indicate loss of read depth of approximately 1%, 3% and 9%, respectively (and relative to 0% loss for control). mtPCR1 buffers for each kit resulted in approximately 11% (control region) and 17% (mtGenome) of bases with no coverage at 130% KCl; adverse effects in read depths were not observed for mtPCR2 of the control region even at the 130% KCl concentration. Reduction of MgSO_4_ to 90% of the control concentration of mtPCR1 of the whole mito genome multiplex resulted in some low-level (0.3%) loss of base calls while maintaining 100% variant detection. Data indicate that when MgSO_4_ was increased to 120% and 130% of the control concentration in the control region assay’s mtPCR1 buffer, 94% and 91% of variants were detected, and 3% and 4% of bases, respectively for these two concentrations, were not detected above default thresholds. 100% variants were detected at each tested MgSO_4_ concentration tested for the mtGenome mtPCR1 buffer, with reduced bases called, relative to the control, at 100%, 110%, 120% and 130%, measured at 0.1%, 0.2%, 0.2% and 0.3%, respectively (Figure S3).

#### 3.8.2. Potential for Differential/Preferential Amplification Among Amplicons

**Figure 5:**
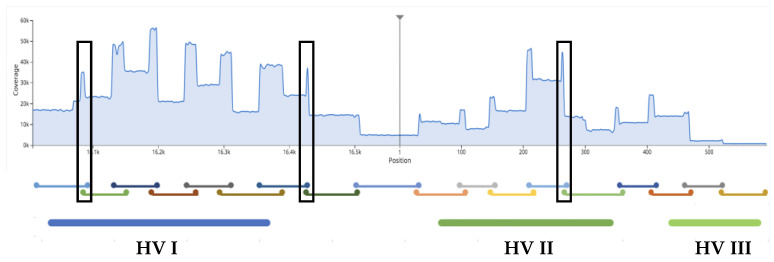
Control region data visualization of Coriell gDNA sample HG03370 as displayed in ForenSeq universal analysis software (UAS) coverage plot (position vs. coverage) with position viewer visible in center at position 0 (vertical line). Tiled amplicons are shown in variously colored horizontal brackets under the coverage plot, as are schematics of hypervariable regions I and II. Three black vertical rectangles show examples of “bat ears” or regions of coverage generated between overlapping amplicons.

The potential exists in nested, tiled PCR for amplification of secondary amplicons along with the targeted, primary amplicons as well as regions that are covered by more than one amplicon (overlapping coverage). These include (1) overlapping coverage (from two to 103 bp) between regions of adjacent amplicons and (2) “doublewide” amplicons, relative to the target region, formed when primers within PCR 1 (from set 1 or from set 2; Figure 1) extend across and include the length of two primary amplicons. Regions of overlap from adjacent amplicons may be viewed in the UAS as “bat ears” (Figure 5). Doublewides can assist in the confirmation of variants that sit under a primer. Because of the ForenSeq mtDNA library preparation approach of employing a PCR 1 for set 1 and one for set 2, small competing amplicons were not observed. Additionally, relative amplicon performance within the mtGenome multiplex was investigated. Though amplicon lengths range from 60 to 209 bp (including primers), amplification of smaller amplicons is not necessarily more efficient than longer amplicons, as shown in Supplemental Figure S2.

#### 3.8.3. Effects of Amplicon Multiplexing

Comparisons of control region variant calling, when 18 control region amplicons were targeted alone and also when targeted in the presence of 541 additional primers in the mtGenome multiplex, were described within the concordance studies described in Section 3.1.1, Section 3.1.2, Section 3.4 and Section 3.5.2 The 122 primers that target the control region are of the same sequences and present at the same ratios in each kit’s primer mix. Average reads per amplicon were assessed as part of repeatability and reproducibility studies, as summarized in Table 4. At the sample plexities used in those studies (48 and 16 libraries per run for control region multiplex and mtGenome, respectively), fewer reads were generated for the whole mtGenome. On average, 2.9X, 11X and 10.7X fold reads per amplicon were observed for the control region multiplex at these maximum recommended sample plexities. Running fewer samples (>8 minimum) assists in increasing read depth; for example, if “no calls” or coverage lower than what is desired are observed.

### 3.9. Species-Specificity

Species-specificity was assessed by virtue of the ability of the control region and mtGenome multiplexes to generate and the UAS to detect, above default analysis parameters, genetic information from nonhuman species. As described in the Materials and Methods, gDNA from eight nonhuman eukaryotes and one prokaryote was processed along with controls (100 pg HL60, NTCs) with each multiplex: 100 pg from two Old World primates (rhesus monkey, cynomolgus monkey), five non-primate mammals (pig, cow, dog, cat, horse), one avian species (domesticated chicken) and *E. coli.* For control region multiplex, samples and controls were amplified in triplicate; these 33 libraries were sequenced with other libraries for a total of 42 sample MiSeq FGx micro sequencing run. The mtGenome multiplex was assessed in a 24 sample plexity standard sequencing run of duplicate libraries of these same nonhuman samples and positive control along with quadruplicate NTCs.

#### 3.9.1. Control Region in the two ForenSeq mtDNA Multiplexes: Species-Specificity

Reads were detected neither in cynomolgus monkey nor in cat libraries (triplicates) from the control region kit; reproducible reads in excess of default UAS analysis settings were detected in five species and ranged from one amplicon (bovine, rhesus) to nine amplicons (equine). The average read counts of these amplicons ranged from 23 (cat) to 85 (equine). A total of 150,247 average reads were detected across the positive HL-60 controls; no reads were detected in the NTCs except for one amplicon with 94 reads, which also had average reads of 23, 37 and 85 in the cat, bovine and equine samples, respectively. A similar assessment was made with control region data from the mtGenome kit; reads were not detected in the duplicate libraries prepared for these same species. The average read count in mtGenome positive controls for the species study was 68,543; no reads were detected in the NTCs. Non-reproducible reads were noted (17–166 reads in one rep only) at a total of six mtDNA amplicons as follows: chicken (five amplicons), *E. coli* (two amplicons)*,* equine (five amplicons) and rhesus (one amplicon).

#### 3.9.2. mtGenome Multiplex: Species-Specificity

**Figure 6:**
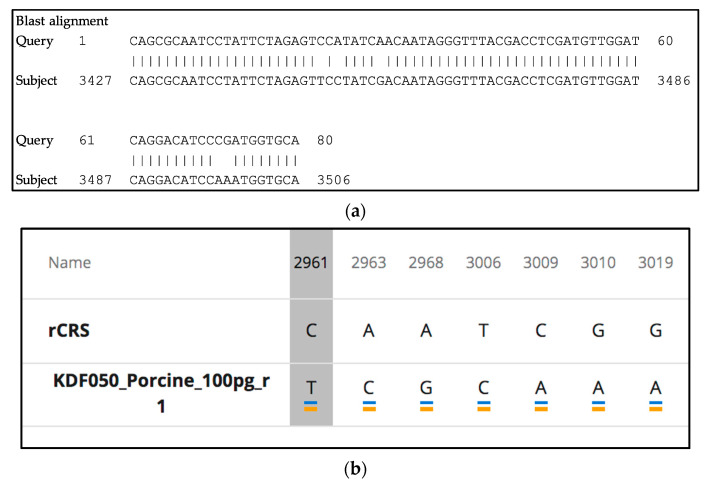
Species-specificity study: (**a**) basic local alignment search tool (BLAST) results for targeted human mtDNA amplicon (query) and porcine genome coordinates (subject, 3427–3506), (**b**) UAS screenshot of variant positions between the rCRS and the one amplicon detected in the replicate porcine libraries.

In the duplicate amplifications for the mtGenome, when assessing data outside of the control region, no reads were detected for the rhesus monkey and the NTCs. Reads were reproducibly detected that align to one and the same human mtDNA amplicon sequence in eight of the nine species tested, with average read counts ranging from 40 (cynomolgus monkey) to 311,325 (porcine) and 68,543 across positive human mtDNA controls. The nonhuman amplicon in these eight species was highly homologous to the human mtDNA MT-RNR2 gene (1671–3229 human positions) that codes for the large 16S rRNA subunit. For example, a query of the human 80 nucleotide target/insert (rCRS sequence), using the online basic local alignment search tool (BLAST), indicated five mismatches between human and porcine sequence ([65,66,67,68,69,70], Figure 6a). In the UAS, this porcine sample had seven reproducible SNVs vs. rCRS, all on this one targeted amplicon and within 58 bases of one another; no other reproducible porcine reads were detected. Approximate amplicon coverage is shown in Supplementary Table S7.

The two monkey species produced six other reproducible amplicons. In addition to the human amplicon described above, four other ForenSeq mtGenome amplicons that spanned the MT-RNR2 gene were detected in each of the two monkey DNA samples with average read counts ranging from 28 to 482 (Supplemental Table S7). The other two amplicons in each monkey DNA sample were highly homologous with tRNA genes as follows: one amplicon with 1261 and 1271 average reads in rhesus and cynomolgus, respectively, align to human mtDNA tRNA genes MT-TA, MT-TN and MT-TW; one amplicon with 9253 and 12,916 average reads in rhesus and cynomolgus, respectively, aligns to human mtDNA tRNA genes MT-TN and MT-C. Cumulatively in cynomolgus monkey, for example, 64 differences were displayed in the UAS relative to the rCRS and were comprised of 14 insertions, 41 SNVs and nine deletions (Supplemental Table S7). Lastly, the rhesus monkey samples produced 105 average reads for one amplicon with homology to the human MT-ND5 gene that codes for NADH dehydrogenase, subunit 5 (complex I). Additional reproducible reads were not detected in the seven other tested species.

### 3.10. Stability in the Presence of Inhibitory Substances

**Figure 7:**
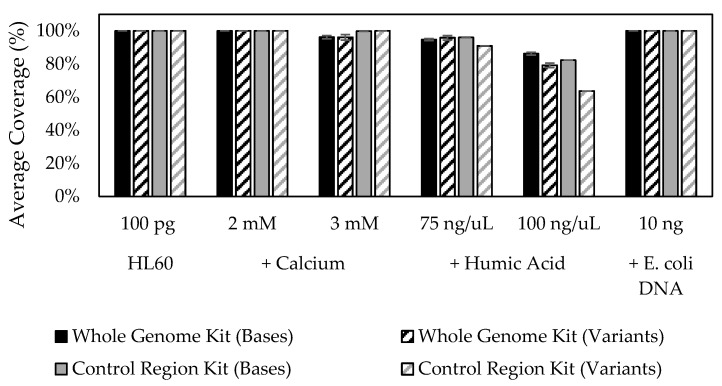
Stability studies: chemical insults and coverage with control region and mtGenome multiplexes. Stability studies were conducted, as described in Materials and Methods, by assessing effects on average coverage (%, *y*-axis) of PCR inhibitors calcium (two concentrations), humic acid (two concentrations) and 10 ng *E. coli* DNA (*x*-axis) of mitochondrial DNA amplification using each ForenSeq mtDNA multiplex and 100 pg of HL60 positive control DNA. Untreated HL60 DNA is shown at the far left, where complete coverage and 100% call rates were observed for each kit. Solid black bars and solid gray bars indicate% bases detected (coverage) above the default analytical threshold in the ForenSeq UAS; hatched black bars and hatched gray bars indicate variant call rates.

Effects of a set of three known PCR inhibitors on ForenSeq mtDNA and MiSeq FGx sequencing efficiency were characterized for the control region and mtGenome kits. Inhibitors, each of which was independently spiked directly into PCR1, and concentrations tested are shown in Figure 7. Read depth (plotted as average coverage (read counts) per base) in samples containing inhibitors were compared to the control sample, to which inhibitors were not added. Control region data indicated similar resistance to PCR inhibition for each kit. Variant call rates were also compared between samples subjected to inhibitors and to untreated positive control DNA. A >95% call rate was observed at all conditions except for 100 ng/µL humic acid, which dropped to 62% for the control region multiplex and to 80% for mtGenome samples (Figure 7). Burrow–Wheeler aligner (BWA; Broad Institute, Cambridge, MA, USA) alignment was used to further analyze samples spiked with *E. coli* DNA for the presence of microbial DNA in sequencing data; bacterial sequences were not detected [60,61].

## 4. Discussion

Next-generation sequencing (NGS), also known as massively parallel sequencing (MPS), simplifies forensic mitotyping workflows and improves overall efficiencies relative to Sanger-type sequencing. ForenSeq PCR-based library preparation kits, universal analysis software and the MiSeq FGx instrument provide end-to-end forensic NGS workflows for human mtDNA analysis. These components were assessed together according to forensic guidelines [1,2]. Two ForenSeq mtDNA kits were developed and validated, one targeting the control region and the other the whole mitochondrial genome (mtGenome). Each kits’ tiled amplicon design promoted complete, unambiguous coverage and maximized on-target reads with low background caused by unintended byproducts by splitting the sample into two PCRs, each of which uses a separate set of staggered primers around the circular molecule, which are pooled prior to sequencing. This design, combined with small amplicons averaging 118 bp and 131 bp for the control region and mtGenome multiplexes, respectively, allows for base coverage and minor variant detection even when partial degradation of the template has occurred, such as with burned or interred bone and other challenging samples. Additionally, when a variant sits under the primer(s) of one primer set, then that variant may be detected reliably in amplicons extended from the companion set. ForenSeq PCR itself is conducted in two-steps: PCR 1 for target amplification and PCR 2 for adapter/index incorporation, which afforded less opportunity for dimer formation and contributed to higher mtDNA sequencing successes than what is produced by library preparation steps combined into one PCR with competing oligonucleotide extension products. The additional prep time can be considered a valuable investment in order to ease downstream data analyses and to recover the maximum information possible from mtDNA.

The MiSeq FGx instrument was designed with forensic science applications in mind, with suggested or required updates provided at a cadence intended to accommodate validated operational casework and databasing. The ForenSeq universal analysis software (UAS) was designed for the analyst, rather than for the bioinformatician per se: the learning curve is slight and regarded as easy to use by multiple forensic testing sites (personal communications). The ForenSeq UAS mtDNA pipeline and tools predicated on the now-defunct, cloud-based mtDNA variant processor and mtDNA variant analyzer applications that were in BaseSpace (Illumina, Inc, San Diego, CA, USA) reside on a stand-alone server thus do not require Internet access. UAS settings are customizable such that lab-specific analysis Methods may easily be created and saved if desired, alongside the preconfigured “default” analysis methods that are provided for each kit. It may be the case that some laboratories wish to employ default analysis method for some types of samples, cases or projects, and could consider lowering one or more thresholds (e.g., minimum read count, analytical threshold, interpretation threshold) if investigating heteroplasmy or interpreting minor mtDNA contributor(s) or other scenarios. Analysis tracking and history logging may be activated in the UAS as desired; sample compare functionality is included as well as strand depth information and reports (e.g., sample, EMPOP and CODIS Reports).

Forensic developmental validation and internal validation studies were conducted in accordance with SWGDAM guidelines. Robustness and limitations were assessed through analysis of a range of environmental and situational variables, including, but not limited to, mock casework and effects of PCR inhibitors, repeatability, reproducibility and concordance studies, sensitivity studies (sample plexity, input DNA amounts) and species cross-reactivity. Performance windows were established and verified for a range of temperatures in thermal cycling as well as a range of concentrations of mtPCR1 and mtPCR2 buffer components around the final, standard concentration for each multiplex. Components were tested individually and included concentrations of as much as +/−30%. Performance windows in which maximal variant detection, base coverage and read depths did not significantly vary were clear, as were defined points where system performance began to deteriorate as with other PCR-based systems, KCl concentration affected amplification efficiency and may correlate with amplicon length [73]. Comparable high-quality results were observed, relative to the standard KCl concentration, at + 10% and as low as −30%, with a measurable reduction in read depth and variant detection noted at +20% and +30% KCl for three of the four mtPCR buffers such that information was lost due to drop out; base calling accuracy was not affected.

Deciding the maximum number of samples to sequence simultaneously (sample plexity) per MiSeq FGx run is dependent upon several factors, including a number of bases targeted (i.e., control region only, entire mtGenome), the capacity of the flow cell used (MiSeq FGx reagent kit “standard” or “micro”), sample quality and desired read depth. Data from high-quality samples indicated that, when using default UAS analysis methods, a maximum of 48 control region libraries (with micro sequencing kit) and 16 mtGenome libraries (with standard sequencing kit) can supply complete coverage and variant calling. In our studies, while complete coverage was achieved for the majority of pristine samples and down to 1 pg input gDNA (Figure 3, for example,) and most challenging samples (human remains, buccal, hair) at these maximum sample plexities, there were instances of coverage loss even from high-quality samples where the control region multiplex did not detect all point heteroplasmy or where the mtGenome multiplex lost coverage of four bases (100 pg) to 356 bases (2 pg). Adjusting sample plexity such that fewer samples and thus increased reads are generated may provide assistance when handling challenging samples by increasing data completeness. For lower quality samples or lower quantity samples, such as <20 pg template, a second optional library purification step, which is a 15-min hands-on time investment, can also increase read depth and variant calling. Using this optional step and running the maximum recommended sample number per run, complete coverage and variant calling were observed due to increased read depths for the control region and for the mtGenome down to 1 pg of input gDNA (default UAS settings; Figure 3a,b). Ultimately, the selection of sample number per run is at the discretion of user laboratories.

Laboratories may desire to sequence mitochondrial DNA of low DNA quantities (e.g., bones and hair shafts) and higher DNA quantities (e.g., family reference samples) together on the same run. In our studies, when mtDNA libraries of disparate quantities and qualities were sequenced together, signal crosstalk was not observed. For example, crosstalk was not detected in control region data from the five sequencing runs with combinations of high input and very low input and/or degraded samples in sensitivity studies (Figure 3a,b), mock casework, mixture analysis or nonhuman species studies. Similarly, in mtGenome studies of reproducibility, mock casework, and mixtures over six sequencing runs with combinations of high input and very low input and/or degraded samples, no crosstalk was observed.

The ability to obtain results from DNA recovered from biological samples subjected to various environmental insults of relevance to mtDNA was investigated. The two ForenSeq mtDNA multiplexes successfully sequenced and called variants from 8 mock casework libraries from dental remains, cremains and environmentally insulted bone samples, rootless hair shafts and buccal swabs. It may be helpful to estimate a degradation index for samples such as these to assist in determining how much extract to add in PCR1 and/or to consider an mtDNA-specific quantification prior to library preparation. Recovery of haplotypes from DNA in the presence of known casework-like levels of PCR inhibitors (chemical insults) that can be co-purified with human remains samples were also evaluated. Of the inhibitors tested (calcium, humic acid, bacterial DNA), none caused a significant reduction in overall read number compared to controls. Conventional troubleshooting approaches for samples that are suspected to be inhibited, such as DNA extract dilution or additional DNA purification, may be helpful for NGS-based testing as well if inhibition is observed.

This report offers a look at the accuracy and the precision of deep mtDNA sequencing of ForenSeq libraries using sequencing by synthesis (SBS). Data generated in reproducibility and repeatability studies of ForenSeq mtDNA assays, the MiSeq FGx and UAS were used to measure variant calling accuracy and precision. Control DNA HL60 has been sequenced hundreds of times (~442) with 100% accuracy and precision in control region variant calling, as well as 100% accuracy across the entire mito-molecule with precision ranging from 97.9% (due to loss of four to 356 base calls) to 100%. For concordance studies, in addition to comparisons to Sanger-type sequencing data, we also used low pass WGS data, which can generate mtDNA sequence simultaneous with nuclear DNA data. Those data are, of course, particularly useful for full mtGenome data assessments; 373 mtGenomes were sequenced as part of this report (>6.18 million bases).

SBS overcomes many limitations of Sanger-type mtDNA sequencing. To provide some context for the experience of implementing a MiSeq FGx System, we note that ForenSeq mtPCR1 required an estimated hands-on time of 15 min followed by 3 h 35 min of thermal cycling and 10 min of hands-on time for mtPCR2 with 90 min of thermal cycling. Library purification and normalization required approximately 45 min of hands-on time and a total of 1 h and 50 min. MiSeq FGx run times were approximately 19 and 28 h for the control region multiplex and the mtGenome multiplex, respectively. Data analysis typically required 1 h in the ForenSeq universal analysis software for a total sequencing and data analysis time of 20–29 h for control region or mtGenome, respectively.

As expected, refined haplogroup assignments were generated when mtGenome variants were used in addition to the control region or HVI and HVII alone. The deeper phylogenic information that can be provided when a contributor’s full mitochondrial genome is considered can bring about actionable investigative leads. In situations where mtGenome data are warranted, improvements relative to Sanger-type sequencing that NGS brings regarding labor, convenience and price, situate forensic mtGenome analyses as much more approachable in an operational setting. For example, 16 high-quality mtGenomes can be generated in a single run, with ~105 min of hand on time.

## Figures and Tables

**Table 1:** Mock casework human remains: control region and mtGenome coverage, variants and European DNA Profiling Group (EDNAP) mtDNA population (EMPOP) haplogrouping, control region concordance between mtDNA multiplexes.

	*Sample Name, Source*	Control Region Multiplex			mtGenome Multiplex			
		*CR Coverage*	*CR Observed Variants*	*Haplogroup*	*mtGenome Coverage*	*mtGenome* *No Call Region(s)*	*mtGenome CR Observed Variants*	*Haplogroup*
CONTEMPORARY SAMPLES	Tooth 1661, InnoGenomics	100%	73G 150T 152C 263G 315.1C 523c 524a 16124C 16223T 16311C 16399G	L3d1b1	99%	5086–5177	73G 150T 152C 263G 315.1C 497M ^1^ 523c 524a 16124C 16223T 16311C 16399G	L3d1b1
	Tooth 1662, InnoGenomics	100%	73G 153G 195C 225A 226C 263G 309.1c 315.1C 16189c ^2^ 16193.1c 16223T 16278T 16519C	X2 + 225	99%	8290–8379	73G 153G 195C 225A 226C 263G 309.1c 315.1C 16189c 16193.1c 16223T 16278T 16519C	X2b4a1
	Tooth 1663, InnoGenomics	100%	73G 150T 152C 195C 198T 263G 315.1C 16189c 16223T 16320T 16519C	L3e2a1	100%		73G 150T 152C 195C 198T 263G 315.1C 16189c 16223T 16320T 16519C	L3e2a1b3
	Tooth 1664, InnoGenomics	100%	73G 146C 152C 195C 263G 309.1C 315.1C 378Y 507C 16223T 16278T 16286T 16294T 16309G 16390A 16519C	L2a1a2	98%	519, 4044–4175, 7216–7367	73G 146C 152C 195C 263G 309.1C 315.1C 378Y 507C 16223T 16278T 16286T 16294T 16309G 16390A 16519C	L2a1a2b
	Tooth 1665, InnoGenomics	100%	64T 93G 185A 189G 200G 236C 247A 263G 315.1C 523a 524c 16129A 16148T 16168T 16172C 16187T 16188G 16189C 16223T 16230G 16311C 16320T 16325C 16362C	L0a1a + 200	97%	4044–4175, 4299–4379, 7021–7182, 7192–7196, 7206, 7216–7367	64T 93G 185A 189G 200G 236C 247A 263G 315.1C 523a 524c 16129A 16148T 16168T 16172C 16187T 16188G 16189C 16223T 16230G 16311C 16320T 16325C 16362C	L0a1a2
	Bone S1, Commercially cremated, SHSU	100%	73G 150T 263G 315.1C 16189c 16193.1c 16270T 16398A	U5b2a2	99%	5307, 5327, 5334–5338, 5343, 6718−6810, 7308−7310, 12,563−12,564, 15,571−15,573	73G 150T 263G 315.1C 16183M ^3^ 16189c 16193.1c 16270T 16398A	U5b2a2b
	Bone S2, embalmed, SHSU	100%	73G 150T 185A 228A 263G 295T 309.1C 315.1C 462T 489C 16069T 16126C	J1c	97%	1103, 1132−1138, 1150, 1164, 1172, 2661−2663, 3606, 5307−5347, 6121, 6139, 6718−6810, 7256−7342, 7508−7559, 11,187−11,189, 12,466−12,614, 15,190, 15,539−15,581	73G 150T 185A 228A 263G 295T 309.1C 315.1C 462T 489C 16069T 16126C	J1c
	Bone S3, embalmed, SHSU	100%	73G 143A 146C 152C 189G 195C 263G 315.1C 16129A 16189c 16192T 16223T 16278T 16294T 16309G 16390A	L2a1	98%	4044−4175, 5081−5177, 5335−5336, 7216−7310	73G 143A 146C 152C 189G 195C 263G 315.1C 16129A 16189c 16192T 16223T 16278T 16294T 16309G 16390A	L2a1n
	Bone S4, SHSU	100%	73G 152C 263G 315.1C 16093Y 16256T 16270T 16399G	U5a1	100%		73G 152C 263G 315.1C 16093Y 16256T 16270T 16399G	U5a1a1b
	Bone S5, burned, SHSU	100%	195C 263G 315.1C 523a 524c	R0	99%	5858−5975, 8444−8446, 12,466−12536, 12,563−12,614	195C 263G 315.1C 523a 524c	H4a1a4b
	Bone S6, burned, SHSU	100%	73G 263G 309.1c 315.1C 16126C 16294T 16296T 16519C	T2	100%	2663, 3550−3606, 5334−5337, 7308−7310, 15,571−15,574	73G 263G 309.1c 315.1C 481Y ^4^ 16126C 16294T 16296T 16519C	T2a1a
	Bone S7, burned, SHSU	100%	263G 309.1c 315.1C 316A 16291T 16519C	H1j2a	100%	15,572	263G 309.1c 309.2c ^5^ 315.1C 316A 16291T 16519C	H1j2a
ANCIENT SAMPLES	Interred bone P2, PSU	100%	73G ^6^ 263G 315.1c ^7^ 489Y ^8^ 16192Y 16256Y 16260Y 16270T 16291T 16399R	U5a1b1	96%	1094−1177, 2668−2671, 3590−3591, 5307−5346, 6109−6141, 6719−6810, 7256−7342, 7545, 8291−8379, 11,193−11197, 12,466−12614, 15,519−15,581	73R ^6^ 263G 315.1C ^7^ 523a ^9^ 16076M ^10^ 16192Y 16256Y 16260Y 16270T 16291T 16399R	U5a1b1c
	Interred bone P43pt1, PSU	100%	152C 263G 309.1c 315.1c 16234T 16270Y	H	99%	5340−5344, 6718−6810, 7314−7318, 15,579	152C 263G 309.1c 315.1c 495Y ^11^ 506Y ^12^ 16234T 16270Y	H13a1d
	Interred bone P48, PSU	100%	257R 263G 315.1C 477C ^13^ 16093Y 16192Y 16270Y 16519C	H1c	99%	5307−5347, 6718−6810, 7258−7266, 7273, 7288−7340, 8345−8349, 12,555−12,559	257R 263G 315.1C 477Y ^13^ 514Y ^14^ 16093Y 16192Y 16270Y 16519C	H1
	Interred bone P73, PSU	100%	73G 153G 195C 263G 309.1C ^15^ 309.2c 315.1C ^17^ 489G 16189c 16223T 16278T 16294T 16519C	X1′2′3	98%	2668−2671, 5307−5347, 6109−6141, 6718−6810, 7256−7348, 12,555−12559, 15,520−15,581	73G 153G 195C 263G 309.1c ^15^ 309.2c 310Y ^16^ 315.1c ^17^ 459Y ^18^ 489G 494Y 496Y 497Y 511Y 513R 514Y 518Y 557Y ^19^ 16188c ^20^ 16189c 16223T 16278T 16294T 16519C	X2

^1^ Base call A present at position 497 (10.4%) in the mtGenome multiplex run was not detected in the control region run; ^2^ base call 16189c should be called 16189C 16193c; ^3^ base call C present at position 16,183 (6.3%; below AT) in control region multiplex run and at 6.3% (above AT) in mtGenome run; ^4^ base call T present at position 481 at 18.7% (above AT) in mtGenome run not detected in control region run ^5^ C insertion present at position 309 at 9.2% (above AT) in mtGenome run not detected in control region run (region had very low read depth); ^6^ base call A present at position 73 at 7.3% (below AT) in control region multiplex run and at 7.5% (above AT) in mtGenome run; ^7^ reference sequence present at position 315 at 10% (above AT) in control region multiplex run and at 3% (below AT) in mtGenome run; ^8^ base call C present at position 489 at 12.8% (above AT) and at 1.9% (below AT) in mtGenome run; ^9^ deletion present at position 523 at 7.8% (below AT) in control region multiplex run and at 6.4% (above AT) in mtGenome run; ^10^ base call A present at position 16,076 at 7.1% (above AT) in mtGenome run not detected in control region run; ^11^ base call T present at position 495 at 1.1% (below AT) in control region multiplex run and at 6.6% (above AT) in mtGenome run; ^12^ base call T present at position 506 at 0.5% (below AT) in control region run and at 7.2% (above AT) in mtGenome run; ^13^ base call T present at position 477 at 6.7% (less than AT) in control region multiplex run and at 9.8% (greater than AT) in mtGenome run; ^14^ base call T present at position 514 at 0.3% (less than AT) in control region multiplex run and at 7.8% (greater than AT) in mtGenome run; ^15^ reference sequence present at position 309 at 4.9% (less than AT) in control region multiplex run and at 11.2% (greater than AT) in mtGenome run; ^16^ base call C present at position 310 at 7.7% (greater than AT) in mtGenome multiplex run not detected in control region (see Section 2.13); ^17^ reference sequence present at position 315 at 6.1% (less than AT) in control region multiplex run and at 10.5% (greater than AT) in mtGenome run; ^18^ low-level mixed base variants from position 459 to 518 at ~14% in mtGenome multiplex run are present in the control region run at ~1% (less than the AT); ^19^ base call T present at position 557 at 7.3% (less than AT) in control region multiplex run and at 6.3% (greater than AT) in mtGenome run; ^20^ reference sequence present at position 16188 at 7.5% (less than AT) in control region multiplex run and at 6.8% (above the AT) in mtGenome run.

**Table 2:** Mock casework: control region and mtGenome coverage, variants and EMPOP haplogrouping, control region concordance between multiplexes and among matched buccal samples and rootless hair shafts from six individuals.

		*Control Region*					*mtGenome*				
*Sample*	*Input*	*CR Coverage*	*CR* *No Call Region(s)*	*CR Observed Variants*	*Haplogroup*	*Concordance* *(Relative to Buccal)*	*mtG Coverage*	*mtGenome* *No Call Region(s)*	*mtG CR Observed Variants*	*Haplogroup*	*Concordance* *(Relative to Buccal)*
Buccal sample 2	100 pg	100%		73G 146C 150T 263G 309.1c 315.1C 523a 524c 16126C 16292T 16294T 16296T 16519C	T2c1 + 146		100%		73G 146C 150T 263G 309.1c 315.1C 523a 524c 16126C 16292T 16294T 16296T 16519C	T2c1e	
0.5 cm Hair sample 2	12 µL	99.9%	310	73G 146C 150T 263G 315.1C 523a 524c 16126C 16292T 16294T 16296T 16519C	T2c1 + 146	100%	100%		73G 146C 150T 263G 309.1c ^1^ 315.1C 523a 524c 16126C 16292T 16294T 16296T 16519C	T2c1e	100%
2 cm Hair sample 2	12 µL	100%		73G 146C 150T 263G 315.1C 523a 524c 16126C 16292T 16294T 16296T 16519C	T2c1 + 146	100%	100%		73G 146C 150T 263G 309.1c ^1^ 315.1C 523a 524c 16126C 16292T 16294T 16296T 16519C	T2c1e	100%
Buccal sample 4	100pg	99.9%	310	146C 263G 309.1C 315.1C 16142T 16325C	HV		99.7%	9538–9590	146C 263G 309.1c ^2^ 309.2c ^3^ 315.1C 16142T 16325C	H47	
0.5 cm Hair sample 4	12 µL	96.9%	303–346	146C 263G 16142T 16325C	HV	100%	98.7%	8290–8379, 9538–9590, 12,496–12,601	146C 263G 309.1c ^2^ 315.1C 16142T 16325C	H47	100%
2 cm Hair sample 4	12 µL	96.3%	303–347	146C 263G 16142T 16325C	HV	100%	99.0%	9541, 9545–9547, 9549–9550, 9552, 9555–9557, 9564, 9568, 9570–9571, 9577, 9581, 9588–9589, 12,466–12,614	146C 263G 309.1c ^2^ 315.1C 16142T 16325C	H47	100%
Buccal sample 5	100pg	100%		263G 315.1C	R0		100%		263G 315.1C	H4a1a1	
0.5 cm Hair sample 5	12 µL	100%		263G 315.1C	R0	100%	100%		263G 315.1C	H4a1a1	100%
2 cm Hair sample 5	12 µL	100%		263G 315.1C	R0	100%	100%		263G 315.1C	H4a1a1	100%
Buccal sample 8	100pg	100%		73G 150T 194T 263G 315.1C 489C 523a 524c 16223T 16362C 16519C	D4b2b2a		100%		73G 150T 194T 263G 315.1C 489C 523a 524c 16223T 16362C 16519C	D4b2b2a	
0.5 cm Hair sample 8	12 µL	100%		73G 150T 194T 263G 315.1C 489C 523a 524c 16223T 16362C 16519C	D4b2b2a	100%	100%		73G 150T 194T 263G 315.1C 489C 523a 524c 16223T 16362C 16519C	D4b2b2a	100%
2 cm Hair sample 8	12 µL	100%		73G 150T 194T 263G 315.1C 489C 523a 524c 16223T 16362C 16519C	D4b2b2a	100%	100%		73G 150T 194T 263G 315.1C 489C 523a 524c 16223T 16362C 16519C	D4b2b2a	100%
Buccal sample 11	100pg	100%		73G 152C 249del 263G 309.1c 315.1C 523a 524c 16108T 16129A 16162G 16172C 16232A 16304C 16357C 16519C	F1a1a		100%		73G 152C 249del 263G 309.1c 315.1C 523a 524c 16108T 16129A 16162G 16172C 16232A 16304C 16357C 16519C	F1a1a	
0.5 cm Hair sample 11	12 µL	96.1%	303–347	73G 152C 249del 263G 523a 524c 16108T 16129A 16162G 16172C 16232A 16304C 16357C 16519C	F1a1a	100%	99.8%	9489–9526	73G 152C 249del 263G 309.1c 315.1C 523a 524c 16108T 16129A 16162G 16172C 16232A 16304C 16357C 16519C	F1a1a	100%
2 cm Hair sample 11	12 µL	99.9%	310	73G 152C 249del 263G 309.1c 315.1C 523a 524c 16108T 16129A 16162G 16172C 16232A 16304C 16357C 16519C	F1a1a	100%	100%		73G 152C 249del 263G 309.1c 315.1C 523a 524c 16108T 16129A 16162G 16172C 16232A 16304C 16357C 16519C	F1a1a	100%
Buccal sample 12	100pg	100%		195Y 263G 309.1c 315.1C ^4^ 16519C	R0		100%		195Y 263G 309.1c 315.1C ^4^ 16519C	H40b	
0.5 cm Hair sample 12	12 µL	96.1%	303–347	195Y 263G 16519C	R0	100%	98.5%	5307, 5311–5312, 5318, 5321, 5323–5332, 6718–6810, 7256–7342, 15,519–15,581	195Y 263G 309.1c 315.1c ^4^ 489Y ^5^ 16519C	H40b	100%
2 cm Hair sample 12	12 µL	100%		195Y 263G 309.1c 315.1C ^4^ 16519C	R0	100%	99.5%	6718–6810	195Y 263G 309.1c 315.1c ^4^ 16519C	H40b	100%

^1^ C insertion at position 309 less than the 10% AT in the control region multiplex run; ^2^ mixed variants 309.1c less than the 10% AT in the control region multiplex run; ^3^ 2nd C Insertion at position 309 less than the 10% AT in the control region multiplex run; ^4^ reference sequence present in control region multiplex run, less than the 10% AT: 6.2% in buccal, 8.1% in 2 cm hair; the reference sequence is present in the mtGenome multiplex run greater than AT: 11% in 0.5 cm hair, 6.2% in 2 cm hair and less than AT at 3.4% in buccal; ^5^ C present at position 489 in 0.5 cm hair at 22% not detected in control region multiplex run nor in buccal or 2 cm hair from individual 12.

**Table 3:** Two-person (2800 M:HL60) mtDNA mixtures at different ratios and DNA inputs: ForenSeq control region and mtGenome multiplexes.

ForenSeq Multiplex	gDNA Input	AT^1^	Mixture Ratio	Expected Variant Allele Ratio (%)	Expected Minor Variant Range (%)	Expected Major Variant Range (%)	Expected Minor Variants (2800 M)	Observed Minor Variants	Minor Variant Detection Rate
Control region	100 pg	3.7%	1:3	25:75	22–36	64–78	10	10	100%
	100 pg	3.7%	1:5	17:83	10–17	82–90	10	10	100%
	100 pg	3.7%	1:15	6:94	4–7	93–96	10	10	100%
	5 pg	3.7%	1:3	25:75	24–36	64–76	10	10	100%
	5 pg	3.7%	1:5	17:83	4–19	81–96	10	11^2^	100%
	5 pg	3.7%	1:15	6:94	3–7	93–97	10	8	80%
mtGenome	100 pg	6%	1:1	50:50	26–47	53–74	27	27	100%
	100 pg	6%	1:3	25:75	11–25	75–89	27	27	100%
	100 pg	6%	1:9	10:90	0–11	89–100	27	15	55.6%
	5 pg	6%	1:1	50:50	24–47	53–76	27	27	100%
	5 pg	6%	1:3	25:75	8–19	81–92	27	27	100%
	5 pg	6%	1:9	10:90	0–8	92–100	27	7	25.9%

^1^ analytical threshold; note: a custom 3.7% AT in ForenSeq UAS was applied for the analysis of control region multiplex in this study, ^2^ unexpected mixed base variant 501 Y was at 4.4%.

**Table 4:** Repeatability and reproducibility studies summary: control region and mtGenome multiplexes.

	Input	Repeatability		Reproducibility	
		Control Region Multiplex	mtGenome Multiplex	Control Region Multiplex	mtGenome Multiplex
**Precision**	2 pg	100%	97.9% ^1^	100%	97.9% ^2^
	20 pg	N/A	99.4% ^3^	100%	N/A
	100 pg	100%	99.98% ^4^	100%	100%
**Concordance**	2 pg	100%	100% ^5^	100%	100% ^6^
	20 pg	N/A	100% ^7^	100.0%	N/A
	100 pg	100%	100% ^8^	100.0%	100% ^9^
**Average reads** **per amplicon**	2 pg	7129	3580	6983	1260
	20 pg	N/A	1764	20,728	N/A
	100 pg	43,401	3146	29,574	3645

Notes: Repeatability and reproducibility were analyzed using 48 samples per multiplex with 16 samples per run across 12 MiSeq FGx runs (three runs each for each multiplex for each study). This generated 56,693 and 79,5312 data points (bases called per mtDNA position) for the control region and the mtGenome, respectively, in repeatability studies, and another 56,693 and 79,5312 data points in reproducibility studies. ^1^ Average loss of coverage of 356 bases for the 2 pg HL60 samples (*n* = 18); ^2^ average loss of coverage of 342 bases for the 2 pg HL60 samples (*n* = 9); ^3^ average loss of coverage of 106 bases for the 20 pg HL60 samples (*n* = 5); ^4^ average loss of coverage of four bases for the 100 pg Coriell samples (*n* = 5); no loss of coverage for 100 pg HL60 samples; ^5^ heteroplasmy: “C” at position 1490, and “A” at position 4821, were not detected at 2 pg in HL60 in all nine replicates; ^6^ heteroplasmy: “C” at position 1490, and “A” at position 4821, were not detected at 2 pg in HL60 in 17 of 18 replicates, or 16 of 18 replicates, respectively. Heteroplasmy was detected at position 1490 at 7% in one replicate; in the two replicates where heteroplasmy at position 4821 occurred, an average of 8% was observed; ^7^ heteroplasmy: “C” at position 1490, and “A” at position 4821, were not detected at 20 pg in HL60 in four of six replicates, or in six of six replicates, respectively. In the two replicates where heteroplasmy at position 1490 occurred, an average of 2.9% was observed; ^8^ heteroplasmy: “A” at position 4821 was not detected at 100 pg in HL60 in six of nine replicates; in the three replicates where heteroplasmy at position 4821 occurred an average of 6% was observed. Heteroplasmy was detected at position 1490 at 4.5% in all nine replicates; ^9^ heteroplasmy: “C” at position 1490 was not detected at 100 pg in HL60 in two of 18 replicates. Heteroplasmy was detected at position 1490 at 5.1% in 16 replicates and at position 4821 at an average of 6.2% for the 18 replicates.

**Table 5:** mtDNA control region concordance studies: control region multiplex vs. whole mtGenome multiplex in five well-characterized DNA samples.

Sample (100 pg)	Expected CR Variants [52,53,54]	Control Region Kit	Whole Genome Kit
		Observed CR Variants (100% Concordance)	Observed CR Variant (100% Concordance)
**CHR**	64Y 73G 195C 204C 207A 263G 309.1C 315.1C 16183C 16189C 16193.1c 16193.2c 16223T 16278T 16519C	64Y 73G 195C 204C 207A 263G 309.1C 315.1C 16183C 16189C 16193.1c 16193.2c 16223T 16278T 16519C	64Y 73G 195C 204C 207A 263G 309.1C 315.1C 16183C 16189C 16193.1c 16193.2c 16223T 16278T 16519C
**9947A**	93G 195C 214G 263G 309.1C 309.2C 315.1C 16311C 16519C	93G 195C 214G 263G 309.1C 309.2C 315.1C 16311C 16519C	93G 195C 214G 263G 309.1C 309.2C 315.1C 16311C 16519C
**HL-60**	73G 150T 152C 263G 295T 315.1C 489C 16069T 16193T 16278T 16362C	73G 150T 152C 263G 295T 315.1C 489C 16069T 16193T 16278T 16362C	73G 150T 152C 263G 295T 315.1C 489C 16069T 16193T 16278T 16362C
**GM03798**	263G 315.1C 16357C 16519C	263G 315.1C 16357C 16519C	263G 315.1C 16357C 16519C
**GM10472A**	73G 185A 228A 263G 295T 315.1C 462T 482C 489C 16069T 16126C 16292T	73G 185A 228A 263G 295T 315.1C 462T 482C 489C 16069T 16126C 16292T	73G 185A 228A 263G 295T 315.1C 462T 482C 489C 16069T 16126C 16292T

**Table 6:** Concordance studies: orthogonal WGS data vs. ForenSeq mtDNA multiplexes, haplogroup assignment using EMPOP.

*Sample*	*Control Region Multiplex: Observed Variants*	*Haplogroup Based on Control Region*	*mtGenome Multiplex: No Call Region(s)*	*mtGenome Multiplex: Control Region Observed Variants*	*Haplogroup Based on mtGenome*	*mtGenome Concordance* *(Compared to 1KPG)*	*CR Concordance* *(Compared to CR Multiplex)*
HG00181	73G 195C 263G 309.1C 315.1C 499A 524.1a 524.2c 16356C 16519C	U4	6922–6988	73G 195C 263G 309.1C 315.1C 499A 524.1a 524.2c 16356C 16519C	U4d1a1	100%	100%
HG00383	263G 315.1C 523a 524c 16093C 16129A 16316G 16519C	H27		263G 315.1C 523a 524c 16093C 16129A 16316G 16519C	H27a	100%	100%
HG00384	73G 150T 263G 309.1c 309.2c 315.1C 16144C 16183M 16189C 16193.1c 16193.2c 16270T	U5b1b1a		73G 150T 263G 309.1c 309.2c 315.1C 16144C 16183M 16189C 16193.1c 16193.2c 16270T	U5b1b1a	100%	100%
HG00844	73G 249del 263G 309.1C 310Y ^1^ 315.1C 489C 16092C 16189C 16193.1c 16193.2c 16223T 16298C 16327T 16355T 16519C	C	470–519, 3550–3606, 13,013–13,080, 15,539–15,581	73G 249del 263G 309.1c 310Y 315.1C 16092C 16189c ^2^ 16193.1c 16223T 16298C 16327T 16355T 16519C	C7a	100%	100%
HG01197	73G 150T 263G 279C 315.1C 455.1T 517T 16224C 16270T	U5b2b3a		73G 150T 263G 279C 315.1C 455.1T 517T 523a 16181R 16224C 16270T	U5b2b3a	100%	100%
HG01204	73G 249del 290del 291del 315.1C 489C 493G 523a 524c 16223T 16298C 16325C 16327T 16519C	C1b2		73G 249del 290del 291del 315.1C 489C 493G 523a 524c 16223T 16298C 16325C 16327T 16519C	C1b2	100%	100%
HG01205	73G 263G 315.1C 523a 524c 16093C 16223T 16278T 16362C 16519C	L3b1a		73G 189R 263G 315.1C 523a 524c 16093C 16223T 16278T 16362C 16519C	L3b1a + @16124	100%	100%
HG01497	73G 263G 309.1C 309.2c 315.1c 498del 499A 524.1a 524.2c 16183c 16189C 16193.1c 16217C 16519C	B2d		73G 263G 309.1C 309.2c 315.1c 498del 499A 524.1a 524.2c 16183c 16189C 16193.1c 16217C 16519C	B2d	100%	100%
HG01498	73G 263G 307c 308c 309c 310c 498del 499A 16182c 16183c 16189C 16193.1c 16217C 16519C	B2d		73G 263G 307c 308c 309c 310c 498del 499A 16182c 16183c 16189C 16193.1c 16217C 16519C	B2d	100%	100%
HG01550	73G 263G 309.1C 309.2c 309.3c 310Y 315.1C 498del 499A 16182C 16183c 16189C 16193.1c 16217C 16519C	B2d		73G 263G 309.1C 309.2c 309.3c 310Y 315.1C 498del 499A 16182c 16183c 16189C 16193.1c 16217C 16519C	B2d	100%	100%
HG01551	73G 150T 263G 315.1C 523a 524c 16051G 16223T 16264T 16519C	L3e4a		73G 150T 263G 315.1C 523a 524c 16051G 16223T 16264T 16519C	L3e4a	100%	100%
HG01790	263G 309.1C 309.2C 315.1C	R0		263G 309.1C 309.2c 315.1C	H33a	100%	100%
HG02190	73G 150T 263G 315.1C 489C 523a 524c 16172C 16182C 16183c 16189C 16193.1c 16223T 16362C 16519C	D5a2		73G 150T 263G 315.1C 489C 523a 524c 16172C 16182c 16183c 16189C 16193.1c 16223T 16362C 16519C	D5a2b	100%	100%
HG02215	263G 315.1C 16311C 16519C	R0		263G 315.1C 16311C 16519C	H3m	100%	100%
HG02236	214G 263G 315.1C 16172C 16519C	HV		214G 263G 315.1C 16172C 16519C	H1	100%	100%
HG02238	263G 309.1C 309.2C 315.1C 16129A 16519C	H		263G 309.1C 309.2c 315.1C 16129A 16519C	H1j1	100%	100%
HG02239	263G 292C 309.1C 315.1C 16519C	R0		263G 292C 309.1c 315.1C 16519C	H1	100%	100%
HG02317	73G 143A 146C 152C 195C 263G 309.1C 315.1C 16129A 16223T 16278T 16294T 16309G 16390A	L2a1c + 16129		73G 143A 146C 152C 195C 263G 309.1C 315.1C 16129A 16223T 16278T 16294T 16309G 16390A	L2a1c5	100%	100%
HG02322	73G 89C 93G 95C 152C 182T 186A 189C 236C 247A 263G 297G 315.1C 316A 523a 524c 16129A 16182C 16183c 16189C 16223T 16235G 16274A 16278T 16293G 16294T 16311C 16360T 16519C	L1c1a2	4044–4175, 7256–7367	73G 89C 93G 95C 152C 182T 186A 189C 236C 247A 263G 297G 315.1C 316A 523a 524c 16129A 16182c 16183c 16189C 16223T 16235G 16274A 16278T 16293G 16294T 16311C 16360T 16519C	L1c1a2	100%	100%
HG02449	73G 150T 263G 273Y 309.1C 315.1C 523a 524c 16051G 16223T 16264T 16519C	L3e4a		73G 150T 263G 273Y 309.1C 315.1C 523a 524c 16051G 16223T 16264T 16519C	L3e4a	100%	100%
HG02450	73G 150T 195C 263G 309.1C 315.1C 499A 16223T 16320T 16399G 16519C	L3e2a1b1		73G 150T 195C 263G 309.1C 315.1C 499A 16223T 16320T 16399G 16519C	L3e2a1b1	100%	100%
HG02513	73G 249del 263G 309.1C 315.1C 521a 522c 523a 524c 16172C 16304C 16465T 16519C	F1a2a		73G 249del 263G 309.1c 315.1C 521a 522c 523a 524c 16172C 16304C 16465T 16519C	F1a2a	100%	100%
HG02521	73G 150T 263G 309.1c 315.1C 16111T 16129A 16223T 16257A 16261T	N9a1		73G 150T 263G 309.1c 315.1C 16111T 16129A 16223T 16257A 16261T	N9a1	100%	100%
HG03369	73G 150T 195C 263G 315.1C 16223T 16265T 16519C	L3e3		73G 150T 195C 263G 315.1C 16223T 16265T 16519C	L3e3b	100%	100%
HG03370	73G 263G 315.1C 372C 523a 524c 16124C 16223T 16278T 16519C	L3		73G 263G 315.1C 372C 523a 524c 16124C 16223T 16278T 16519C	L3b1a	100%	100%
HG03372	73G 150T 195C 263G 315.1C 16223T 16265T 16519C	L3e3		73G 150T 195C 263G 315.1C 16223T 16265T 16519C	L3e3b	100%	100%
HG03577	73G 150T 195C 263G 309.1C 315.1C 16177G 16223T 16311C 16320T 16354T 16519C	L3e2		73G 150T 195C 263G 309.1C 315.1C 16177G 16223T 16311C 16320T 16354T 16519C	L3e2a	100%	100%
HG03578	73G 146C 152C 195C 263G 315.1C 524.1a 524.2c 524.3a 524.4c 16223T 16233G 16278T 16294T 16309G 16368C 16390A 16519C	L2a1a1		73G 146C 152C 195C 263G 315.1C 524.1a 524.2c 524.3a 524.4c 16223T 16233G 16278T 16294T 16309G 16368C 16390A 16519C	L2a1a1	100%	100%
HG03583	73G 189C 195C 263G 315.1C 523del 524c 16126C 16179T 16215G 16223T 16256A 16284G 16311C	L3h1b1a		73G 189C 195C 263G 315.1C 523del 524c 16126C 16179T 16215G 16223T 16256A 16284G 16311C	L3h1b1a	100%	100%
HG03594	16T 73G 93G 188G 200G 204C 263G 309.1C 315.1C 489C 16153A 16223T 16287T 16327A 16519C	M91a		16T 73G 93G 188G 200G 204C 263G 309.1C 315.1C 489C 16153A 16223T 16287T 16327A 16519C	M91a	100%	100%
HG03595	41T 73G 153G 263G 309.1C 315.1C 489C 16223T 16234T 16295G 16311C 16320T 16519C	M		41T 73G 153G 263G 309.1C 315.1C 489C 16223T 16234T 16295G 16311C 16320T 16519C	M49	100%	100%
HG03600	73G 195A 263G 315.1C 489C 523a 524c 16179del 16223T 16519C	M30		73G 195A 263G 315.1C 489C 523a 524c 16179del 16223T 16519C	M30d1	100%	100%
NA12812	44.1C 263G 309.1C 309.2C 315.1C 16093C 16129A 16183C 16189C 16193.1c 16519C	HV		44.1C 263G 309.1C 309.2C 315.1C 16093C 16129A 16183C 16189C 16193.1c 16519C	H1 + 16189	100%	100%
NA12813	73G 263G 309.1C 315.1C 16114A 16129A 16189c 16192Y 16192.1t 16256T 16270T 16294T 16526A	U5a2a		73G 263G 309.1C 315.1C 16114A 16129A 16189c 16192Y 16192.1t 16256T 16270T 16294T 16526A	U5a2a	100%	100%
NA12814	73G 263G 315.1C 16192T 16256T 16270T 16291T 16399G	U5a1b1		73G 263G 315.1C 16192T 16256T 16270T 16291T 16399G	U5a1b1a2	100%	100%
NA12815	73G 263G 315.1C 16129A 16316G 16519C	H		73G 263G 315.1C 16129A 16316G 16519C	H27	100%	100%
NA12872	263G 309.1C 309.2c 315.1C 16172C 16311C	HV		263G 309.1C 309.2c 315.1C 16172C 16311C	HV6	100%	100%
NA12873	152C 195C 263G 309.1c 309.2c 315.1C 16293G 16311C 16525G	H11a6		152C 195C 263G 309.1c 309.2c 315.1C 16293G 16311C 16525G	H11a6	100%	100%
NA12874	73G 185A 188G 228A 263G 295T 309.1C 315.1C 462T 489C 16069T 16126C 16319A	J1c		73G 185A 188G 228A 263G 295T 309.1C 315.1C 462T 489C 523a 16069T 16126C 16319A	J1c8a	100%	100%
NA19240	73G 150T 152C 195C 263G 315.1C 16172C 16183c 16189C 16193.1c 16223T 16293T 16320T 16519C	L3e2b		73G 150T 152C 195C 263G 315.1C 16172C 16183c 16189C 16193.1c 16223T 16293T 16320T 16519C	L3e2b5	100%	100%
NA20346	73G 150T 195C 263G 315.1C 16145A 16172C 16189c 16193.1c 16193.2c 16223T 16320T 16519C	L3e2b		73G 150T 195C 263G 315.1C 16145A 16172C 16189c 16193.1c 16193.2c 16223T 16320T 16519C	L3e2b1a1	100%	100%
NA20356	73G 263G 309.1c 315.1C 16172C 16219G 16278T 16291Y 16519C	U6a		73G 263G 309.1c 315.1C 16172C 16219G 16278T 16291Y 16519C	U6a5	100%	100%
NA20509	263G 309.1C 309.2C 309.3c 310Y 315.1C 523a 524c 16182C 16183c 16189C 16193.1c 16261T 16274A 16356C 16519C	H1b		263G 309.1C 309.2C 309.3c 315.1C 523a 524c 16182c 16183c 16189C 16193.1c 16193.2c 16261T 16274A 16356C 16519C	H1b1	100%	100%
NA20510	73G 189G 195C 204C 207A 263G 315.1C 16192T 16223T 16309G 16325C 16519C	W6		73G 189G 195C 204C 207A 263G 315.1C 16192T 16223T 16309G 16325C 16519C	W6	100%	100%
NA20828	73G 263G 309.1c 315.1C 497T 524.1a 524.2c 524.3a 524.4c 16129A 16177G 16224C 16311C 16390A 16519C	K1a12a1a		73G 263G 309.1c 315.1C 497T 524.1a 524.2c 524.3a 524.4c 16129A 16177G 16224C 16311C 16390A 16519C	K1a4f1	100%	100%
NA20832	146C 263G 309.1C 309.2c 315.1C 16519C	HV		146C 263G 309.1C 309.2c 315.1C 16519C	H1n	100%	100%
NA20845	73G 152C 263G 309.1c 315.1C 489C 16086C 16129A 16223T 16519C	M		73G 152C 263G 309.1c 315.1C 489C 16086C 16129A 16223T 16519C	M5a2a	100%	100%
NA21143	73G 146C 263G 309.1C 309.2c 315.1C 489C 16129A 16223T 16320T	M		73G 146C 263G 309.1C 309.2c 315.1C 489C 16129A 16223T 16320T	M5c1	100%	100%
NA21144	73G 195C 263G 315.1C 16093C 16519C	R8		73G 195C 263G 315.1C 16093C 16519C	R8a1b	100%	100%

^1^ See Section 2.13 regarding 310Y; ^2^ see Section 2.13 regarding 16189c.

**Table 7:** Lineage distribution of 48,882 sequences addressed in ForenSeq mtDNA primer design [46].

L Lineages “African”			M Lineages “Asian”			N Lineages “Eurasian”		
**hg**	#	**%**	**hg**	#	**%**	**hg**	#	**%**
L3	2135	35.6%	M	5250	50%	H	9167	28%
L0	1500	25%	D	2358	22%	U	4231	13%
L2	1322	22%	C	1651	16%	B	4193	13%
L1	878	14.7%	E	456	4%	J	2319	7%
L4	105	1.8%	G	437	4%	T	2237	7%
L5	39	0.7%	Z	191	2%	K	1817	6%
L6	12	0.2%	Q	177	2%	F	1663	5%
Total	5991	100%	Total	10,520	100%	A	1386	4%
Overall 12% (5991/48,882)			Overall 22% (10,520/48,882)			R	1077	3%
						N	785	2%
						HV	735	2%
						I	718	2%
						V	693	2%
						W	529	2%
						X	470	1%
						P	159	0.5%
						Y	135	0.4%
						S	49	0.2%
						O	8	0.02%
						Total	32,371	100%
						Overall 66% (32,371/48,882)		

Notes: “hg” denotes haplogroup, “#” is the total number of mtGenomes in the database for each category.

## Data Availability

The data presented in this study are available within this article, tables, figures and supplemental tables and figures.

## Supplementary Materials

The following are available online at https://www.mdpi.com/article/10.3390/genes12040599/s1, Figure S1: Screen shot of the sample representation plot from the ForenSeq universal analysis software 2.0/2.1 for the control region multiplex HL60 gDNA dilution series, Table S1: Purified gDNA samples obtained from Coriell Institute for Medical Research used in this study, Table S2A: Sensitivity studies design summary, Table S2B: Mixture study design summary, Table S2C: Repeatability study design summary, Table S2D: Reproducibility study design summary, Table S2E: Sample multiplexing study design summary, Table S3: mtGenome variants detected in the mock casework human remains from the project report generated in the ForenSeq universal analysis software, Table S4: mtGenome variants detected in the mock casework matched buccal and rootless hair shafts from the project report generated in the ForenSeq universal analysis software, Table S5: mtGenome variants detected in five well characterized DNA samples from the project report generated in the ForenSeq universal analysis software, Table S6: mtGenome variants detected in 1000 genomes DNA samples from the project report generated in the ForenSeq universal analysis software, Table S7: Approximate amplicon coverage for samples in the species-specificity study with the mtGenome multiplex.
