# Supplementary material for: Human Mitochondrial Control Region and mtGenome: Design and Forensic Validation of NGS Multiplexes, Sequencing and Analytical Software
Source: Genes (Basel). 2021 Apr 19;12(4):599. doi: 10.3390/genes12040599 (PMC8073089; doi:10.3390/genes12040599)
Supplement: Supplementary file 1 [file genes-12-00599-s001.zip › genes-1070304 supp Figs.pdf]

Supplementary Material Figure 1

(a)

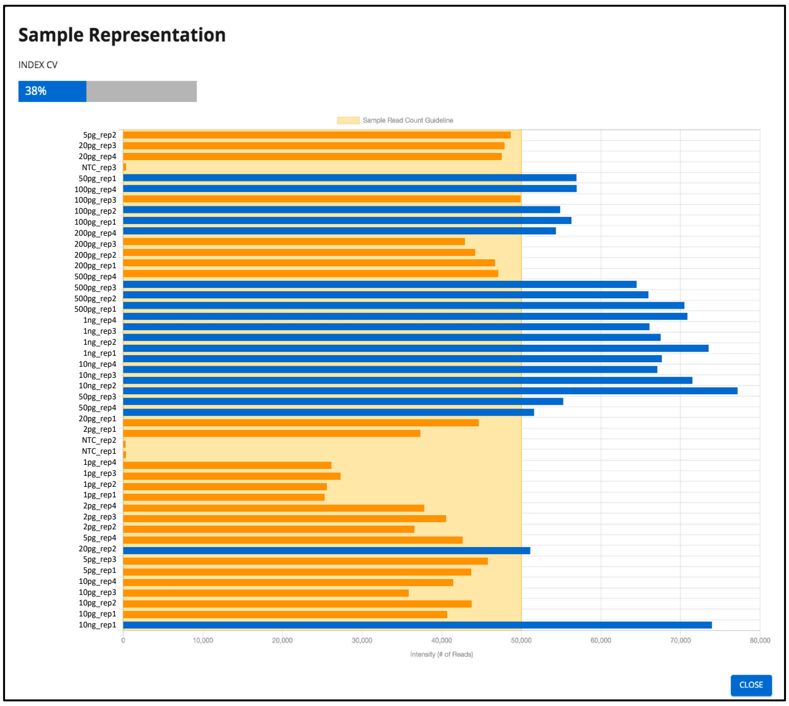

(b)

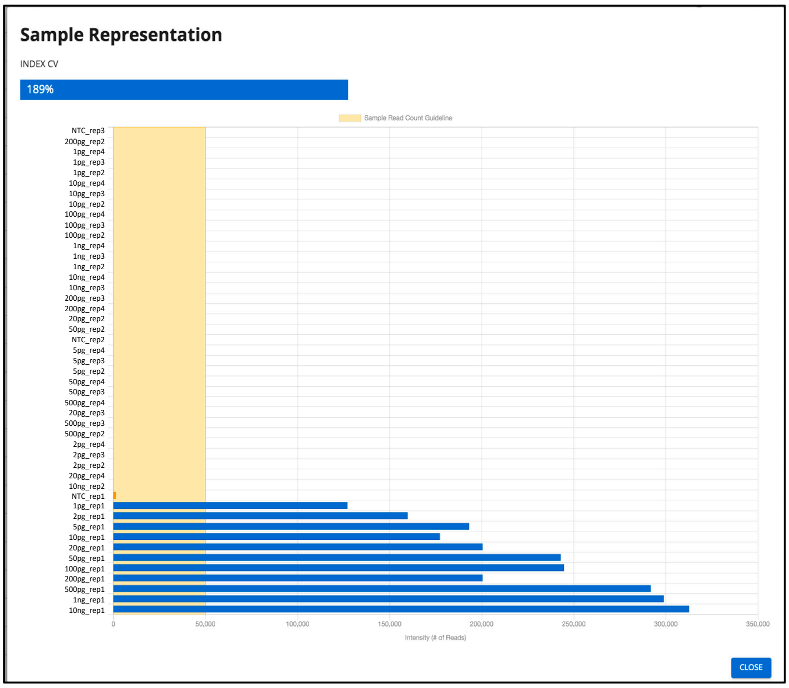

(c)

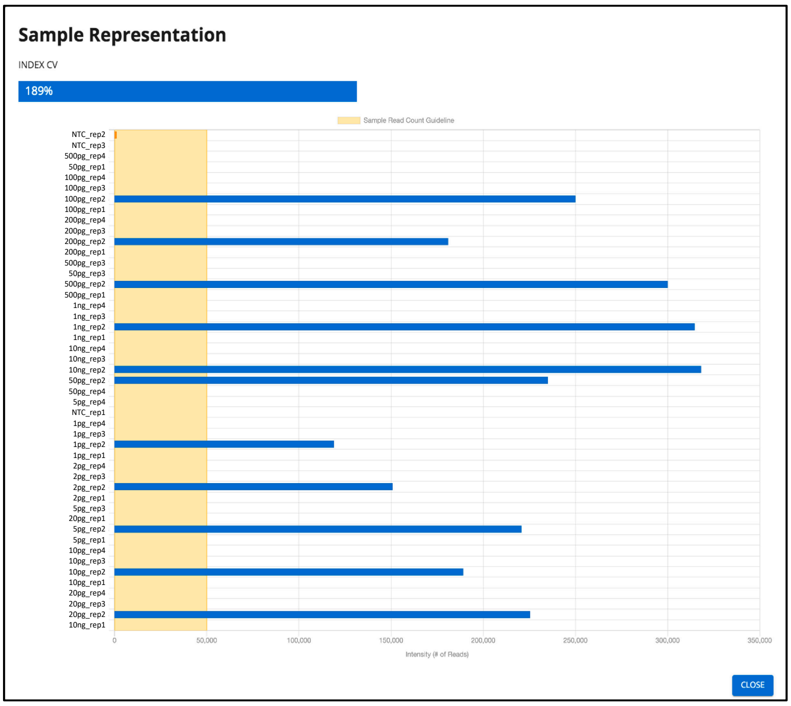

Figure S1. Screen shot of the Sample Representation plot from ForenSeq Universal Analysis Software 2.0 for the control region multiplex HL60 gDNA dilution series. The samples names are shown (y-axis) and the number of paired reads (Intensity) for each sample is indicated by the bars (x-axis). The sample order is random on these plots for each run. Sample Representation plot, for the 47 sample plexity run shown in (a), shows reads for all samples including NTCs. Sample Representation plots for the two 12 sample plexity runs performed immediately after the 47 sample plexity run, as shown in (b) and (c), have reads detected only for the samples on the runs with no reads detected for samples potentially carried over from the previous runs.

Supplementary Material Figure 2

(a)

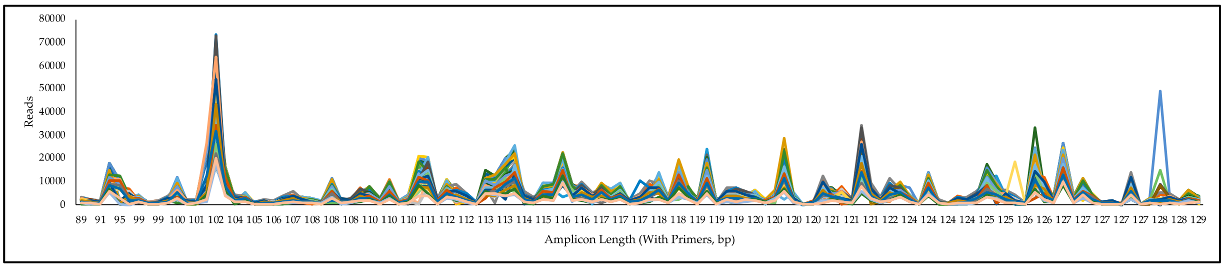

(b)

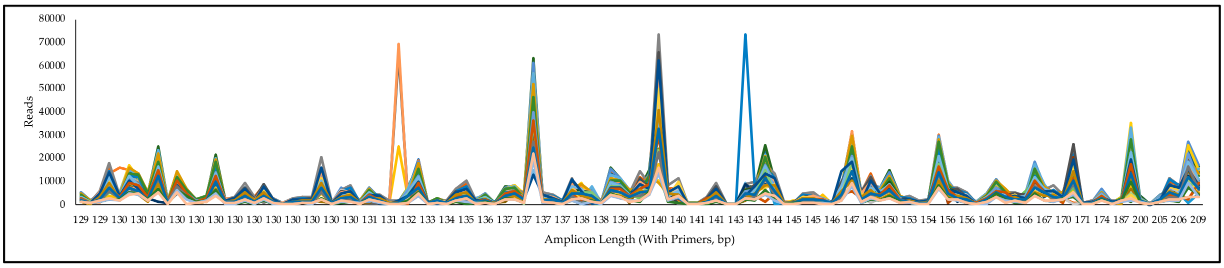

(c)

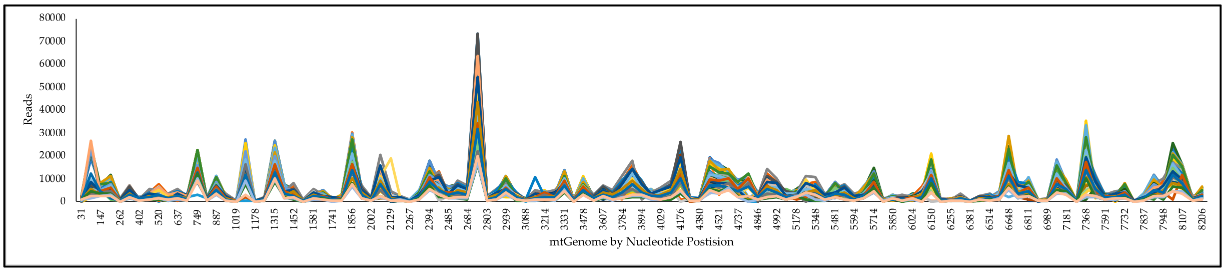

(d)

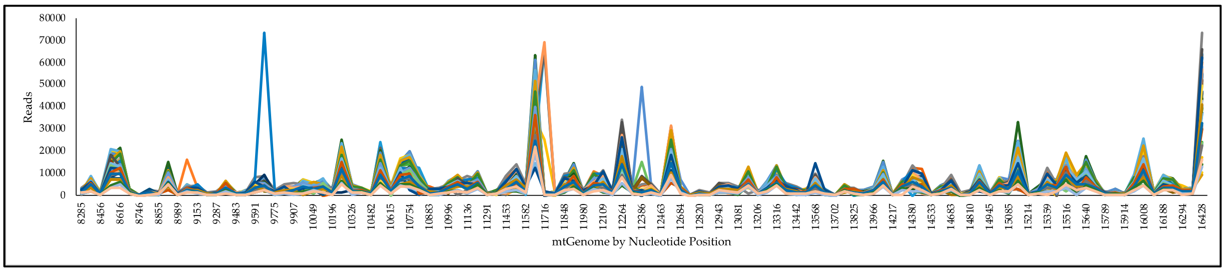

Figure S2. Amplicon coverage for 49 Coriell DNA mtGenome samples included in the concordance and orthogonal methods study (Table 6). Reads were determined for amplicons and sorted based on length (a, b) or based on position in the mtGenome (c, d).

Supplementary Material Figure 3.

(a)

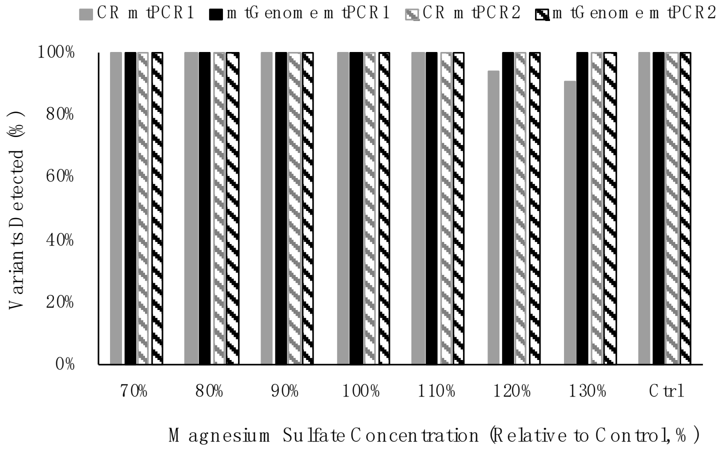

(b)

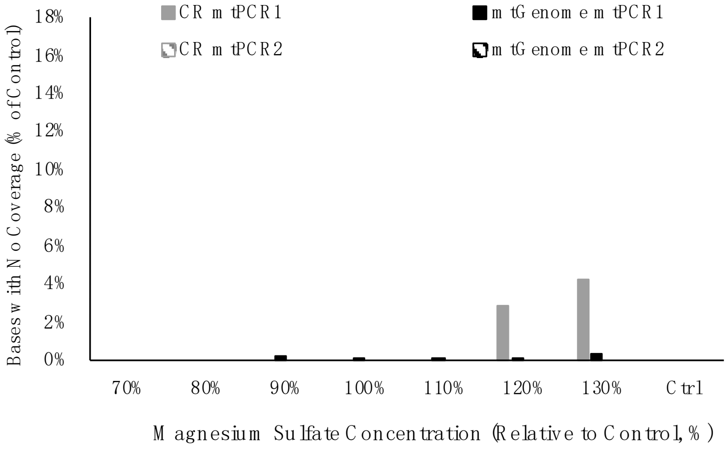

(c)

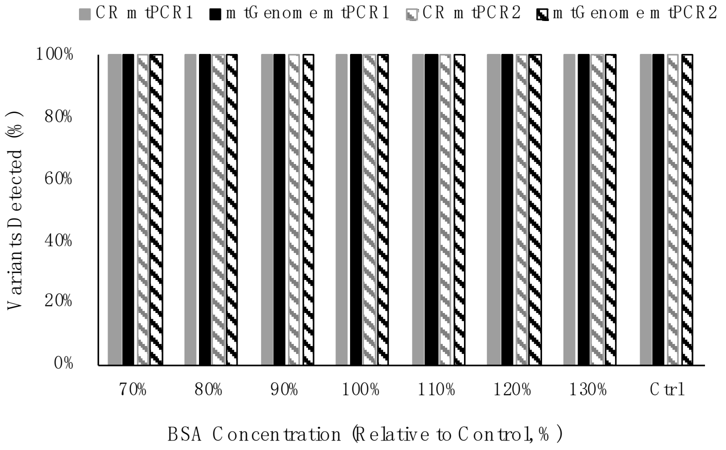

(d)

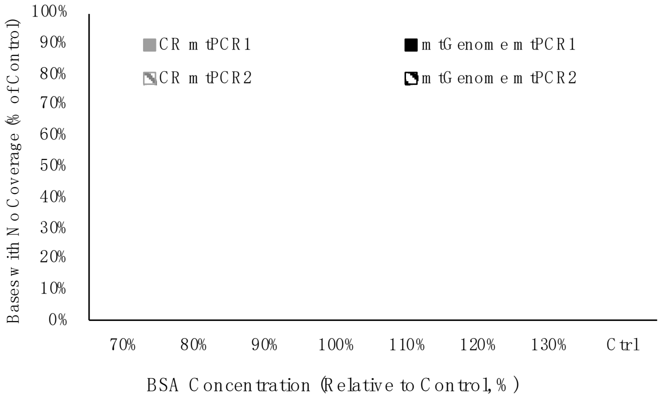

(e)

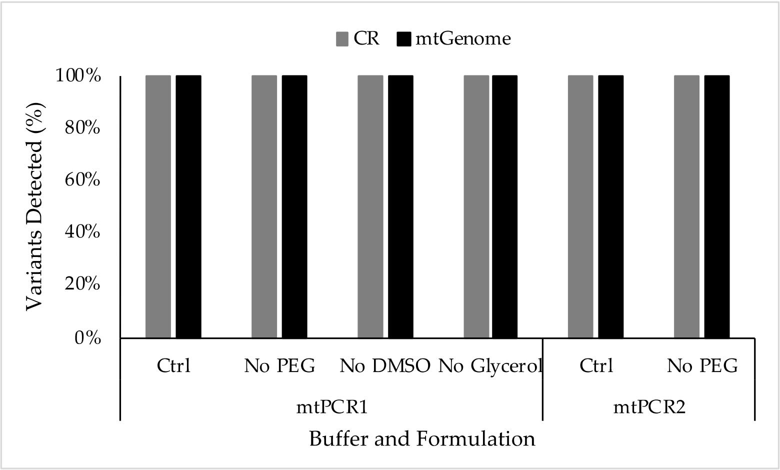

(f)

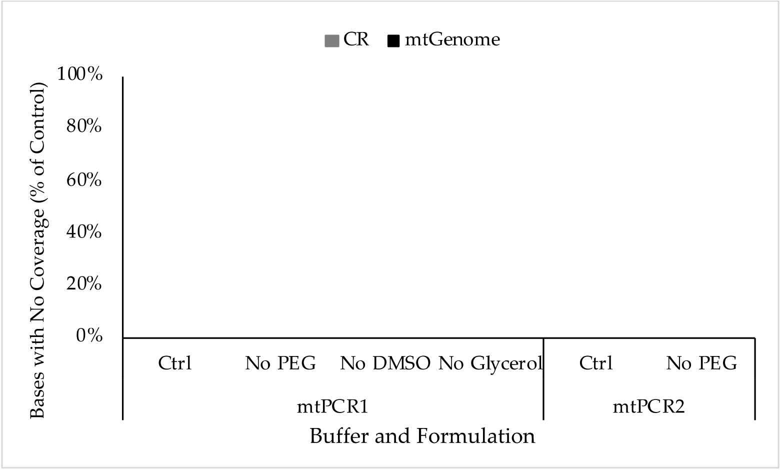

Figure S3. Assessment of critical reagents in PCR-based studies: magnesium sulfate, BSA, PEG, DMSO and glycerol concentration effects on variant detection and coverage in the control region and mtGenome multiplexes, relative to controls. Effects of increased and decreased $MgSO_4$ concentrations on the four mtDNA PCR buffers

(mtPCR1 and mtPCR2 for each kit; x-axes in (a) and (b)) on variants detected (% relative to total; y-axis in (a)) and on bases with no coverage (% relative to control; y-axis in (b)) were assessed using 100 pg of HL60 positive control gDNA. 100% $MgSO_4$ is the titration control, and "Ctrl" is the commercial buffer lot of mtPCR1 and mtPCR2 for each multiplexes. Increased $MgSO_4$ relative to the control, and thus the manufactured standard, can contribute to low data loss when 48 and 16 libraries per sequencing run for the control region (using MiSeq FGx Micro Kit) and whole mito genome libraries (using MiSeq FGx Kit), respectively, are run and data analyses employ default software settings. No effects were observed for varied BSA concentrations on the four mtDNA PCR buffers (mtPCR1 and mtPCR2 for each kit; x-axes in (c) and (d)) on variants detected (% relative to total; y-axis in (c)) and on bases with no coverage (% relative to control; y-axis in (d)) as assessed using 100 pg of HL60 positive control gDNA. 100% BSA is the titration control, and "Ctrl" is the commercial buffer lot of mtPCR1 and mtPCR2 for both multiplexes. No effects were observed for no PEG, DMSO or glycerol on the four mtDNA PCR buffers (mtPCR1 and mtPCR2 for each kit; x-axes in (e) and (f)) on variants detected (% relative to total; y-axis in (e)) and on bases with no coverage (% relative to control; y-axis in (f)) as assessed using 100 pg of HL60 positive control gDNA. "Ctrl" is the commercial buffer lot of mtPCR1 and mtPCR2 for both multiplexes.
